# Mortality study of civilian employees exposed to contaminated drinking water at USMC Base Camp Lejeune: a retrospective cohort study

**DOI:** 10.1186/1476-069X-13-68

**Published:** 2014-08-13

**Authors:** Frank J Bove, Perri Zeitz Ruckart, Morris Maslia, Theodore C Larson

**Affiliations:** 1 Agency for Toxic Substances and Disease Registry (ATSDR), Division of Toxicology and Human Health Sciences, 4770 Buford Highway, MS F-58, Atlanta, GA 30341, USA; 2 ATSDR, Division of Community Health Investigations, 4770 Buford Highway, MS F-59, Atlanta, GA 30341, USA

**Keywords:** Mortality, Cancers, Trichloroethylene, Tetrachloroethylene, Vinyl chloride, Benzene, Drinking water

## Abstract

**Background:**

Two drinking water systems at U.S. Marine Corps Base Camp Lejeune, North Carolina were contaminated with solvents during 1950s-1985.

**Methods:**

We conducted a retrospective cohort mortality study of 4,647 civilian, full-time workers employed at Camp Lejeune during 1973–1985 and potentially exposed to contaminated drinking water. We selected a comparison cohort of 4,690 Camp Pendleton workers employed during 1973–1985 and unexposed to contaminated drinking water. Mortality follow-up period was 1979-2008. Cause-specific standardized mortality ratios utilized U.S. age-, sex-, race-, and calendar period-specific mortality rates as reference. We used survival analysis to compare mortality rates between Camp Lejeune and Camp Pendleton workers and assess the effects of estimated cumulative contaminant exposures within the Camp Lejeune cohort. Ground water contaminant fate/transport and distribution system models provided monthly estimated contaminant levels in drinking water serving workplaces at Camp Lejeune. The confidence interval (CI) indicated precision of effect estimates.

**Results:**

Compared to Camp Pendleton, Camp Lejeune workers had mortality hazard ratios (HRs) >1.50 for kidney cancer (HR = 1.92, 95% CI: 0.58, 6.34), leukemias (HR = 1.59, 95% CI: 0.66, 3.84), multiple myeloma (HR = 1.84, 95% CI: 0.45, 7.58), rectal cancer (HR = 1.65, 95% CI: 0.36, 7.44), oral cavity cancers (HR = 1.93, 95% CI: 0.34, 10.81), and Parkinson’s disease (HR = 3.13, 95% CI: 0.76, 12.81). Within the Camp Lejeune cohort, monotonic exposure-response relationships were observed for leukemia and vinyl chloride and PCE, with mortality HRs at the high exposure category of 1.72 (95% CI: 0.33, 8.83) and 1.82 (95% CI: 0.36, 9.32), respectively. Cumulative exposures were above the median for most deaths from cancers of the kidney, esophagus, rectum, prostate, and Parkinson’s disease, but small numbers precluded evaluation of exposure-response relationships.

**Conclusion:**

The study found elevated HRs in the Camp Lejeune cohort for several causes of death including cancers of the kidney, rectum, oral cavity, leukemias, multiple myeloma, and Parkinson’s disease. Only 14% of the Camp Lejeune cohort died by end of follow-up, producing small numbers of cause-specific deaths and wide CIs. Additional follow-up would be necessary to comprehensively assess drinking water exposure effects at the base.

## Background

United States Marine Corps (USMC) Base Camp Lejeune is located in Onslow County, North Carolina. Samples taken during 1980 through 1985 at Camp Lejeune detected solvents in drinking water supplied by the Hadnot Point (HP) treatment plant serving the main area (“mainside”) of the base where most workplaces were located. The HP supply wells were contaminated by an on-base landfill used for chemical dumping as well as underground storage tank leaks and waste disposal practices at the base’s industrial area [1]. The highly contaminated HP supply wells were shut down by early February 1985.

The primary contaminant in the HP distribution system was trichloroethylene (TCE) with a maximum detected level of 1,400 micrograms per liter (μg/L). The maximum level of tetrachloroethylene (PCE) in the HP drinking water was 100 μg/L and benzene was also detected. Trans-1,2-dichloroethylene (DCE) and vinyl chloride were present in the HP system due to the degradation of TCE in ground water [1].

Between 20 and 30 supply wells were operating in the HP system at any one time since the system began operation in 1942 [1]. Water from all the supply wells serving the HP system was mixed together at the treatment plant prior to distribution. A majority of the supply wells in the HP system were not contaminated, so contamination levels varied depending on the wells in use at a particular time [1].

Current U.S. maximum contaminant levels (MCLs) for TCE, PCE and benzene are 5 μg/L; the MCL for vinyl chloride is 2 μg/L; and the MCL for DCE is 100 μg/L. TCE has recently been classified as a human carcinogen [2-4]. Vinyl chloride and benzene are also classified as human carcinogens [5]. PCE is classified as a “likely” or “probable” human carcinogen [3,6,7].

Several meta-analyses and reviews have assessed the effects of these chemicals on cancers and other chronic diseases [2-7]. Most of the evidence has come from occupational studies where the primary route of exposure was inhalation. On the other hand, drinking water exposure to these chemicals usually involves contributions to total internal body dose from three routes: ingestion, inhalation and dermal. The dose from the inhalation and dermal routes may be as high as the dose from the ingestion route. For example, an internal dose via inhalation to TCE during a 10-minute shower may equal the internal dose via the ingestion of 2 liters of TCE-contaminated drinking water [8]. If a worker at Camp Lejeune consumed cold tap water at his/her workplace, then the route of exposure would be primarily via ingestion. However, if a worker used hot water at the workplace, for example, in tea or coffee, washing hands, or showering (e.g., after exercising or at the end of the shift), then the inhalation and dermal routes of exposure would be important.

The literature is limited on health effects of drinking water exposures to these chemicals. A drinking water study in New Jersey observed associations between TCE and the incidence of leukemia and non-Hodgkin lymphoma (NHL), and between PCE and NHL incidence [9]. PCE-contaminated drinking water was associated with the incidence of lung cancer, bladder cancer, leukemia, rectal cancer, and female breast cancer in a study at Cape Cod, MA [10-12]. No studies have evaluated associations between drinking water exposures to these chemicals and medically confirmed, non-cancer diseases in adults.

The purpose of this study was to determine whether potential exposures of employees to contaminated drinking water at Camp Lejeune increased risk of mortality from cancers and other chronic diseases.

## Methods

We identified *a priori* several diseases of primary interest: cancers of the kidney, hematopoietic system (NHL, leukemia, multiple myeloma, Hodgkin lymphoma), liver, bladder, esophagus and cervix. Kidney cancer, NHL and liver cancer were selected because the U.S. Environmental Protection Agency (EPA) and the International Agency For Research On Cancer cited evidence for a causal association with TCE exposure, although the evidence for liver cancer is “more limited” than the evidence for kidney cancer and NHL [2-4]. The National Toxicology Program (NTP) concluded that there was “evidence for consistent positive associations” between PCE and esophageal and cervical cancer, and EPA cited evidence for associations between PCE and bladder cancer, NHL, and multiple myeloma [3,5-7]. Benzene is a known cause of leukemia.

Diseases of secondary interest were identified *a priori* based on information from literature reviews suggesting possible associations with the contaminants or with solvents in general: aplastic anemia, amyotrophic lateral sclerosis (ALS), multiple sclerosis (MS), kidney and liver diseases, Parkinson’s disease, and cancers of the connective tissue, brain, pancreas, oral cavity, pharynx, lung, larynx, prostate, breast, colon and rectum [2,4-7,13-15]. Because this was a data linkage study with no smoking information, we evaluated smoking-related diseases not known to be associated with the contaminants to assess possible confounding: cardiovascular disease, chronic obstructive pulmonary disease (COPD) which includes emphysema and chronic bronchitis, and stomach cancer.

### Study population and eligibility

The Camp Lejeune cohort consisted of 4,647 full time civilian employees who began working at the base anytime between April 1973 and December 1985. A comparison cohort from USMC Base Camp Pendleton consisted of 4,690 full time civilian employees who met the same criteria, but were not employed at Camp Lejeune during April 1973-December 1985. Camp Pendleton is located along the Southern California coast in northern San Diego County and southern Orange County. Both bases had similar types of occupations but Camp Pendleton did not have a contaminated drinking water supply [16].

We obtained the Defense Manpower Data Center (DMDC) quarterly personnel files for employees at Camp Lejeune and Camp Pendleton. The DMDC began data collection in the last quarter of 1972. There was a gap in the dataset for the first quarter of 1973 and the quarterly data resumed continuously from the second quarter of 1973 onward. Because we had no information on the employment history of those who were employed at either base prior to 1973, we limited the study to those who were not included in the DMDC dataset for the last quarter of 1972 but who were in the dataset anytime from April 1973 through December 1985. We assumed that those not in the DMDC dataset in the last quarter of 1972 were first employed at either base on or after 1973. Personnel transaction codes indicating changes in employment status (e.g., hiring, promotions, retirement) were available in the DMDC dataset beginning in the second quarter of 1974 but could not be used to determine employment start dates because of missing data and coding problems.

For each individual, the quarterly DMDC data contained full name (starting in the last quarter of 1981), Social Security number (SSN), location of employment (city, state, and zip codes), date of birth, sex, race/ethnicity, highest education level attained, paygrade, and occupation code. This study was approved by the Centers for Disease Control and Prevention Institutional Review Board.

### Vital status ascertainment

Personal identifier information from the DMDC database (i.e., name when available, SSN, date of birth, and sex) was matched using a customized algorithm to data in the Social Security Administration (SSA) Death Master File (DMF) and SSA Office of Research, Evaluation and Statistics (ORES) Presumed Living Search to determine vital status [17,18]. Of the combined Camp Lejeune and Camp Pendleton cohorts, almost 50% could not be uniquely matched to the ORES file or their vital status was listed as “unknown” in the ORES file. For these individuals, a commercial tracing service was used to obtain information on their vital status. Identified deaths and individuals whose vital status remained unknown were then searched in the National Death Index (NDI). Those whose vital status remained unknown after the NDI search were considered “lost to follow-up” but contributed person-years to the study until the last date they were included in our DMDC database or the last date they were known to be alive based on the commercial tracing service information. Underlying and contributing causes of death information were obtained from the NDI Plus.

### Exposure assessment

Due to the limited number of historical drinking water samples for volatile organic compounds, the Agency for Toxic Substances and Disease Registry (ATSDR) conducted a historical reconstruction of the spatial and temporal distribution of the contaminants. Details of the methodology and results have been summarized in a peer-reviewed published report [1]. Briefly, we used ground water fate and transport and distribution system models to compute monthly average estimates of the concentrations of the contaminants in the Hadnot Point distribution system [1]. The estimated monthly average concentrations of contaminants in the Hadnot Point water system increased over time during 1973–1985 (Table 1).

**Table 1:** Estimated Monthly Average Contaminant Concentrations in the Hadnot Point system, 1973 – 1985

April 1973* –January 1985**					
**Contaminant**	**Mean (μg/L)**	**Median (μg/L)**	**Range (μg/L)**	**# Months > MCL**	**# Months >100 μg/L**
Tetrachloroethylene	14.9	14.5	0 – 38.7	114	0
Trichloroethylene	355.5	356.6	30.9 – 783.3	142	127
Vinyl Chloride	23.3	20.3	1.0 – 67.3	140	0
Benzene	5.2	4.1	0 – 12.2	63	0
April 1973* – December 1979					
Tetrachloroethylene	9.7	9.6	0 – 24.1	56	0
Trichloroethylene	280.4	274.1	30.9 – 546.3	81	69
Vinyl Chloride	14.7	14.3	1.0 – 33.4	79	0
Benzene	3.3	3.2	0 – 5.8	4	0
January 1980 – January 1985*					
Tetrachloroethylene	21.8	21.8	2.2 – 38.7	58	0
Trichloroethylene	455.2	449.1	42.6 – 783.3	61	58
Vinyl Chloride	34.7	36.0	4.2 – 67.3	61	0
Benzene	7.7	7.6	1.6 – 12.2	59	0

*First quarter of continuous DMDC quarterly personnel data on DOD employees.

**Contaminated wells were shut down in February 1985. From March through December 1985, estimated monthly average levels of trichloroethylene, tetrachloroethylene and vinyl chloride were <1 μg/L, and benzene was <4 μg/L.

Virtually all civilian workers at Camp Lejeune resided off-base. The contamination at Camp Lejeune did not affect off-base drinking water supplies. Exposure to the contaminated drinking water would occur only when the civilians were at work at the base. The mainside area of the base contained maintenance shops, administrative offices, commissaries, storage yards and warehouses. Most of the workplaces were located at mainside. Therefore, we assumed that most civilian workers at Camp Lejeune spent the major portion of their workday in the mainside area, which was served by the Hadnot Point water system. We also assumed that workers at Camp Lejeune were exposed to contaminated drinking water via consumption and/or other uses while at their workplaces during the workday. Since this was a data linkage study, we did not have information on water usage by the workers at Camp Lejeune. For example, we had no information on ingestion or whether the workers showered after their shift or during exercise breaks on base.

We assigned the estimated monthly average contaminant concentrations in the Hadnot Point drinking water to each employee during the period of employment at Camp Lejeune. The median length of employment during 1973–1985 for employees in the Camp Lejeune cohort was about 2.5 years.

### Data analysis

Follow-up began on January 1, 1979 (when NDI began data collection) or the start of employment at either Camp Lejeune or Camp Pendleton, whichever was later. Follow-up continued until the end of the study period, December 31, 2008, if the person was known to be alive, or to the date of death. Those with unknown vital status were followed until the last date they were known to be alive based on available data. We used IBM SPSS Statistics 20 for data manipulation and data management and SAS 9.3 for data analyses.

We used the Life Table Analysis System (LTAS) to compute cause-specific, standardized mortality ratios (SMRs) and 95% confidence intervals comparing the Camp Lejeune and Camp Pendleton cohorts to the age- sex- race-and calendar period-specific U.S. mortality rates for underlying and multiple (contributing) causes of death [19].

We could not calculate SMRs for aplastic anemia because LTAS combined aplastic anemia with “anemias of other and unspecified type”. SMRs also could not be calculated for specific leukemias because LTAS combines the leukemias. LTAS also combines liver cancers with cancers of the biliary passages and gall bladder, therefore a separate SMR for liver cancer could not be calculated.

#### a) Comparisons between Camp Lejeune and Camp Pendleton cohorts

We used Cox extended regression models with age as the time variable and base location as a time-varying dichotomous variable to calculate hazard ratios (HRs) comparing mortality rates between the Camp Lejeune and Camp Pendleton cohorts [20]. These analyses avoided a possible “healthy worker effect” bias which occurs when comparing mortality rates in relatively healthy workers to the U.S. mortality rates for cancers and other chronic diseases [21].

We accounted for a “latency period” by lagging exposure to a base by 10, 15, and 20 years in addition to an analysis with no lag. For example, a 10 year lag would assign to an individual aged 29, the base the individual was employed at age 19. If this individual was not yet employed at age 19, then the person-year for age 29 was assigned to a category “not employed at either base”. We used the Akaike’s information criterion (AIC), a measure of model goodness of fit, to select an appropriate lag period.

Supplementary analyses were conducted comparing the Camp Lejeune cohort to the Camp Pendleton cohort stratified by sex, by “white” race, and by occupation (blue collar vs white collar).

#### b) Analyses within the Camp Lejeune cohort

Within the Camp Lejeune cohort, we evaluated estimated exposure-response relationships between cumulative exposures to drinking water contaminants and cause of mortality using Cox extended regression models with age as the time variable and cumulative exposure as a time-varying variable. Estimated monthly average contaminant concentrations in the Hadnot Point water system and the dates of employment at Camp Lejeune were used to calculate cumulative exposures (“μg/L-years”) to each contaminant and to the total amount of these contaminants (“TVOC”).

We evaluated cumulative exposures as continuous variables, both untransformed and using the log base 10 transformation. The log transform of cumulative exposure can capture exposure-response relationships in which the response plateaus or attenuates at higher levels of cumulative exposure (Steenland and Deddens 2004). We added a small constant, 0.1 μg/L-years, to the log transformed cumulative exposure to avoid taking the logarithm of zero [22]. A one unit increase in the log-transformed cumulative exposure variable corresponds to a ten-fold increase in cumulative exposure. We restricted the analyses of the continuous cumulative exposure variables to diseases with at least 5 deaths in the Camp Lejeune cohort.

We also categorized cumulative exposures into tertiles and dichotomous (above or below the median) variables based on the cohort’s distribution of maximum cumulative exposure. Because of small numbers resulting in HRs of zero or infinity, some of the causes of death could not be evaluated using the tertile and/or dichotomous categorization.

The cumulative exposure analyses focused on PCE, TCE, vinyl chloride, benzene and TVOC. Because cumulative exposures to the contaminants were correlated, making it difficult to distinguish which contaminant might have caused an association with a disease, each Cox regression model included only one contaminant at a time or TVOC.

We accounted for a latency period between the drinking water exposures and the occurrence of death by lagging the exposure over a specified period. We assessed exposure lag periods of 10, 15, and 20 years as well as a “no lag” period. For example, when a 10-year exposure lag was used, an individual at age 29 would be assigned a cumulative exposure level the individual experienced as of age 19. We used the AIC value to select an appropriate exposure lag period.

The use of either categorical or continuous exposure variables (whether transformed or not) imposes a structure on the exposure-response relationship which may be inaccurate [22]. To obtain a more flexible, smoothed exposure-response curve, we specified a restricted cubic spline (RCS) function for cumulative exposure in the Cox extended model [23]. For the analysis of each contaminant, knots were located at the 5^th^, 50^th^, and 95^th^ percentiles among those with any cumulative exposure to the contaminant. We selected these percentiles because they were symmetric for the distribution of those with any cumulative exposure to the contaminant and encompassed most of the range of cumulative exposures [22,23]. Placing the knots at these percentiles also separated those with very low cumulative exposure and those with very high cumulative exposure from the rest of the distribution. (Splines using knots at the 10^th^, 50^th^, and 90^th^ percentiles and at the 20^th^, 50^th^ and 80^th^ percentiles were also explored, but the shape of the HR curves did not differ appreciably from splines with knots at the 5^th^, 50^th^, and 95^th^ percentiles.) The RCS function allowed the shape of the HR curve to vary within and between these knots and restricted the curve to be linear before the first knot and after the last knot. The resulting curve is useful for assessing whether the exposure-response relationship is adequately captured by either the categorical or continuous exposure variables. Splines were restricted to those diseases with at least 10 deaths.

In subsequent analyses, we evaluated duration at Camp Lejeune and duration exposed to contaminated drinking water as time-varying categorical variables. We assessed exposure intensity by computing time-independent, continuous and categorical variables for average exposure.

#### c) Confounder assessment

DMDC and NDI data were available for sex, race, date of death, age at death, paygrade, education level, and occupation. For confounding to occur, a risk factor must be associated with the exposure as well as with the disease of interest. To identify potential confounding, we used a “10% change in the estimate” rule [24]. Final Cox extended models included sex, race, occupation (blue collar vs white collar), and education level.

Information on smoking, alcohol consumption, and occupational history prior to or after employment at either base, was not available from the databases used in this study. We evaluated the magnitude of possible smoking confounding by subtracting the log HR among smoking-related diseases from the log HR of the disease of interest [25].

#### d) Interpretation of findings

Interpretation of study findings was based on the magnitude of the adjusted SMR or HR. For analyses internal to the Camp Lejeune cohort, we also based our interpretation on the exposure-response relationship, giving more emphasis to monotonic trends in the categorical cumulative exposure variables. A monotonic trend occurs when every change in the HR with increasing category of exposure is in the same direction, although the trend could have flat segments but never reverse direction [26]. Because exposure-response trends could be distorted by biases such as exposure misclassification, we also emphasized non-monotonic exposure-response trends when an elevated HR was observed in the high exposure group.

We computed 95% confidence intervals to show the precision of the HR and regression coefficient estimates, and we included p-values for informational purposes only. We did not use statistical significance testing to interpret findings [26-30].

## Results

The Camp Lejeune and Camp Pendleton cohorts were similar on type of occupation, number of months employed at either base, and percent with at least a high school education, but differed somewhat on race and sex (Table 2). Slightly over one-third of both cohorts were employed at their bases during the study period (1973–1985) for one year or less. About 37% of the Camp Lejeune cohort and 33% of the Camp Pendleton cohort were employed at their bases for more than 4 years during the study period.

**Table 2:** Demographics of the Camp Lejeune and Camp Pendleton cohorts

**Factor**	**Camp Lejeune**	**Camp Pendleton**
	**N = 4,647**	**N = 4,690**
Male	42.8%	49.3%
Female	57.2%	50.7%
“white”	82.2%	78.8%
African American	15.4%	9.0%
“other” or unknown	2.4%	12.3%
Median age, start of follow-up	31	34
Median age, end of follow-up	58	60
% ≥65 yrs, end of follow-up	28.1%	37.4%
Not a high school graduate	6.8%	3.9%
High school graduate	70.6%	84.5%
College graduate	22.6%	11.7%
White Collar	69.7%	64.8%
Blue Collar	30.3%	35.2%
% Construction Trades	15.1%	12.5%
% Maintenance/Mechanics	8.7%	12.7%
% Firefighters	2.5%	3.9%
% Laundry Workers	1.3%	1.0%
% Vehicle/Equipment Operation	1.7%	2.2%
% Other	1.0%	2.9%
% Occupation as painter	1.1%	1.0%
% Worked with solvents	27.1%	27.7%
% Worked in food preparation	1.1%	2.2%
Median months employed, 1973-1985	29	27
Total Deaths	654 (14.1%)	869 (18.5%)
% deaths occurring >1995	69.4%	67.0%
Total lost to follow-up	62 (1.3%)	95 (2.0%)
Total person-years of follow-up	123,659	123,065

Both cohorts had similar median ages at the start and end of follow-up but differed somewhat on the percent sixty-five and older at the end of follow-up. Both cohorts were relatively young with a substantial majority under the age of 65 at the end of follow-up.

In the Camp Lejeune and Camp Pendleton cohorts, 654 deaths (14.1%) and 869 deaths (18.5%) occurred respectively. Vital status at the end of follow-up was unknown for ≤2% in the cohorts, and these individuals were lost to follow-up after their last date in our DMDC database or last date that information was available from the SSA or commercial tracing service.

### Standardized Mortality Ratio (SMR) analyses

We found the results for the contributing (or multiple) causes of death to be similar to the results for the underlying cause of death, so only the results for underlying cause of death are shown. Comparing each cohort to the U.S. mortality rates, we observed that the majority of the SMRs were less than 1.00, indicating a healthy worker effect for cancers and non-cancers (Table 3). For the diseases of primary interest, we observed SMRs above 1.00 in the Camp Lejeune cohort for kidney cancer (SMR = 1.30, 95% CI: 0.52, 2.67) and the hematopoietic cancers (SMR = 1.15, 95% CI: 0.74, 1.71), in particular, leukemias and multiple myeloma (SMR = 1.55, 95% CI: 0.80, 2.71; and SMR = 1.50, 95% CI: 0.55, 3.28, respectively). Leukemias were also elevated in the Camp Pendleton cohort (SMR = 1.33, 95% CI: 0.72, 2.22) as was liver cancer (SMR = 1.12, 95% CI: 0.56, 2.00). Of the diseases of secondary interest, both the Camp Lejeune and Camp Pendleton cohorts had SMRs > 1.00 for cancers of the brain and pancreas. Other causes of death with SMRs > 1.00 included ALS in the Camp Pendleton cohort, and Parkinson’s disease and cancer of the larynx, lung, prostate and rectum in the Camp Lejeune cohort. There were no deaths from male breast cancer at either base.

**Table 3:** Standardized Mortality Ratios (SMRs), Underlying cause of death

**Underlying Cause of Death**	**Camp Pendleton (reference)**			**Camp Lejeune**		
	**Obs.**	**Exp.**	**SMR (95% CI)**	**Obs.**	**Exp.**	**SMR (95% CI)**
All Causes	869	1,084	0.80 (0.75, 0.86)	654	765	0.86 (0.79, 0.92)
All Cancers	257	322	0.80 (0.70, 0.90)	229	237	0.97 (0.84, 1.10)
**Diseases of Primary Interest**						
Kidney Cancer	6	7.27	0.82 (0.30, 1.80)	7	5.40	1.30 (0.52, 2.67)
Bladder Cancer	4	5.78	0.69 (0.19, 1.77)	2	3.76	0.53 (0.06, 1.92)
Liver* Cancer	11	9.84	1.12 (0.56, 2.00)	3	7.23	0.42 (0.09, 1.21)
Esophageal Cancer	8	8.78	0.91 (0.39, 1.80)	4	6.21	0.64 (0.18, 1.65)
Cervical Cancer	0	2.94	0 (0.00, 1.25)	1	2.98	0.34 (0.01, 1.87)
Hematopoietic Cancers	25	28.83	0.87 (0.56, 1.28)	24	20.89	1.15 (0.74, 1.71)
Hodgkin Lymphoma	0	0.96	0.00 (0.00, 3.86)	1	0.83	1.20 (0.03, 6.69)
NHL**	8	11.50	0.70 (0.30, 1.37)	5	8.34	0.60 (0.19, 1.40)
Multiple Myeloma	3	5.81	0.52 (0.11, 1.51)	6	3.99	1.50 (0.55, 3.28)
Leukemias	14	10.56	1.33 (0.72, 2.22)	12	7.73	1.55 (0.80, 2.71)
**Diseases of Secondary Interest**						
Pancreatic Cancer	22	16.39	1.34 (0.84, 2.03)	12	11.77	1.02 (0.53, 1.78)
Colon Cancer	13	25.32	0.51 (0.27, 0.88)	12	17.61	0.68 (0.35, 1.19)
Rectal Cancer	4	5.07	0.79 (0.22, 2.02)	4	3.76	1.06 (0.29, 2.72)
Soft Tissue Cancers	2	2.10	0.95 (0.12, 3.44)	1	1.75	0.57 (0.01, 3.19)
Brain Cancer	9	7.94	1.13 (0.52, 2.15)	7	6.68	1.05 (0.42, 2.16)
Laryngeal Cancer	1	3.09	0.32 (0.01, 1.80)	4	2.16	1.85 (0.50, 4.74)
Lung*** Cancer	82	101.60	0.81 (0.64, 1.00)	80	73.20	1.09 (0.87, 1.36)
Oral Cancers****	2	6.15	0.33 (0.04, 1.18)	4	4.43	0.90 (0.25, 2.31)
Breast (female) Cancer	14	23.46	0.60 (0.33, 1.00)	21	21.42	0.98 (0.61, 1.50)
Prostate Cancer	12	15.65	0.77 (0.40, 1.34)	10	9.16	1.09 (0.52, 2.01)
Liver Diseases	19	22.64	0.84 (0.50, 1.31)	9	18.85	0.48 (0.22, 0.91)
Kidney Diseases	7	13.98	0.50 (0.22, 1.00)	7	9.00	0.78 (0.34, 1.54)
ALS	4	2.96	1.35 (0.37, 3.46)	1	2.29	0.44 (0.01, 2.44)
Multiple Sclerosis	1	1.92	0.52 (0.01, 2.91)	1	1.89	0.53 (0.01, 2.95)
Parkinson’s Disease	4	4.54	0.88 (0.24, 2.26)	5	2.28	2.19 (0.71, 5.11)
**Smoking-related Diseases (not known to be related to solvent exposure)**						
Stomach Cancer	7	7.88	0.89 (0.36, 1.83)	4	5.50	0.73 (0.20, 1.86)
Cardiovascular Disease†	317	380.45	0.83 (0.75, 0.93)	210	244.37	0.86 (0.75, 0.98)
COPD	47	47.29	0.99 (0.73, 1.32)	40	29.99	1.33 (0.95, 1.82)

*Biliary passages, liver and gall bladder ** Non-Hodgkin Lymphoma.

***Trachea, bronchus, and lung ****Buccal cavity and Pharynx.

†Includes diseases of the heart and other diseases of the circulatory system.

Camp Lejeune = 4,647; person-years = 123,659.

Camp Pendleton = 4,690; person-years = 123,065.

Of the smoking related diseases not known to be related to solvent exposure, only COPD was elevated in the Camp Lejeune cohort (SMR = 1.33, 95% CI: 0.95, 1.82).

### Comparison of Camp Lejeune with Camp Pendleton

We used Cox extended regression models with age as the time variable to compare the mortality rates in the Camp Lejeune cohort with the Camp Pendleton cohort (Table 4). A 10-year lag of person-years at a base was selected because it had a slightly lower AIC value compared to other lags and no lag, and the HRs were adjusted for sex, race, education and occupation (blue collar vs white collar).

**Table 4:** Camp Lejeune vs Camp Pendleton: Hazard ratios and 95% confidence intervals, adjusted by sex, race, occupation (blue collar vs white collar) and education, 10-year lag

**Underlying Cause of Death**	**Hazard Ratio**	**95% LCL**	**95% UCL**	**p-value**	**CL #**	**CP #**
All Cancers	1.12	0.92	1.36	0.27	197	234
**Diseases of Primary Interest**						
Kidney Cancer	1.92	0.58	6.34	0.28	7	5
Bladder Cancer	0.65	0.12	3.65	0.62	2	4
Liver* Cancer	0.62	0.16	2.45	0.49	8	10
Esophageal Cancer	0.58	0.15	2.22	0.43	3	8
Hematopoietic Cancers	1.40	0.76	2.59	0.28	22	23
Non-Hodgkin Lymphoma	0.83	0.26	2.67	0.76	5	8
Multiple Myeloma	1.84	0.45	7.58	0.40	6	3
Leukemias	1.59	0.66	3.84	0.30	10	12
**Diseases of Secondary Interest**						
Pancreatic Cancer	0.54	0.24	1.20	0.13	9	21
Colorectal Cancers	1.14	0.54	2.39	0.73	14	16
Colon Cancer	1.01	0.43	2.38	0.98	10	13
Rectal Cancer	1.65	0.36	7.44	0.52	4	3
Brain Cancer	0.65	0.21	2.04	0.46	5	8
Lung** Cancer	1.25	0.89	1.75	0.19	69	74
Oral Cancers***	1.93	0.34	10.81	0.46	4	2
Breast (female) Cancer	1.21	0.58	2.51	0.61	18	14
Prostate Cancer	1.17	0.49	2.82	0.72	10	12
Liver Diseases	0.87	0.34	2.25	0.78	8	10
Kidney Diseases	1.23	0.39	3.87	0.72	6	7
Parkinson’s Disease	3.13	0.76	12.86	0.11	5	4
**Smoking-related Diseases (not known to be related to solvent exposure)**						
Stomach Cancer	0.71	0.17	2.96	0.64	3	6
Cardiovascular Disease†	0.93	0.77	1.13	0.46	185	288
COPD	1.21	0.78	1.88	0.40	38	46

Diseases not evaluated due to small numbers include: laryngeal cancer, Hodgkin lymphoma, cervical cancer, soft tissue cancers, multiple sclerosis, ALS, and aplastic anemia.

CL #: number of deaths in the Camp Lejeune cohort.

CP #: number of deaths in the Camp Pendleton cohort.

LCL: lower confidence limit UCL: upper confidence limit.

*Biliary passages, liver and gall bladder **Trachea, bronchus, and lung.

***Buccal cavity and Pharynx.

†Includes heart diseases and other diseases of the circulatory system.

Camp Lejeune had an elevated HR for “all cancers” (HR = 1.12, 95% CI: 0.92, 14.36). Of the diseases of primary interest, Camp Lejeune had elevated HRs for kidney cancer (HR = 1.92, 95% CI:0.58, 6.34) and hematopoietic cancers (HR = 1.40, 95% CI: 0.76, 2.59), in particular multiple myeloma (HR = 1.84, 95% CI:0.45, 7.58) and leukemias (HR = 1.59, 95% CI:0.66, 3.84).

Each cohort had 6 deaths due to acute nonlymphocytic leukemia (ANLL) but less than 5 deaths due to each of the other leukemia subgroups. The HR for ANLL was 2.13 (95% CI: .57, 7.95) when Camp Lejeune was compared to Camp Pendleton.

No other diseases of primary interest were elevated in the Camp Lejeune cohort. Because there were only 3 deaths due to the combined grouping of cancers of the liver, gall bladder and biliary passages in the Camp Lejeune cohort, we did not evaluate liver cancer separately. Hodgkin lymphoma and cervical cancer could not be evaluated because there was only 1 death in the Camp Lejeune cohort and no deaths in the Camp Pendleton cohort.

Diseases of secondary interest that had HRs > 1.50 included Parkinson’s disease (HR = 3.13, 95% CI:0.76, 12.86), oral cavity cancers (HR = 1.93, 95% CI: 0.34, 10.81), and rectal cancer (HR = 1.65, 95% CI: 0.36, 7.44). Not evaluated due to small numbers (<2 deaths at either base) were aplastic anemia (one death at Camp Lejeune only), multiple sclerosis, laryngeal cancer, and cervical cancer. For most of the causes of death, the confidence intervals for the HRs were wide because of small numbers of deaths.

Supplementary analyses stratified by sex, race, and occupation (blue collar vs white collar) were conducted (Additional file 1: Tables S3a-c). The elevated HRs for the hematopoietic cancers were observed among males. Leukemias were elevated among blue collar workers but not white collar workers. Five of the 10 deaths due to prostate cancer in the Camp Lejeune cohort were African Americans whereas there were no deaths among African Americans in the Camp Pendleton cohort.

Among the smoking-related diseases not known to be associated with solvent exposure, only COPD was elevated in the Camp Lejeune cohort with HR of 1.21. Using the HR for COPD to adjust for the possible confounding effects of smoking would reduce the HRs for the diseases of primary and secondary interest by approximately 17.5%. Some diseases of secondary interest that were also smoking-related diseases, such as lung cancer and oral cancers, were elevated in the Camp Lejeune cohort, indicating possible confounding by smoking. However, HRs for other smoking-related diseases such as cardiovascular disease, and cancers of the bladder, esophagus, stomach, pancreas, and liver were <1.0 in the Camp Lejeune cohort, indicating no confounding by smoking.

### Analyses internal to the Camp Lejeune cohort

To assess whether there was an exposure-response relationship between estimated cumulative exposure (“μg/L –years”) to each of the contaminants, (and total contaminants, “TVOC”) and cause of death, analyses were restricted to the Camp Lejeune cohort. Cumulative exposure was evaluated as an untransformed and transformed (log base 10) continuous variable (Additional file 2: Tables S1a-b) as well as categorized into tertiles and dichotomous variables (Additional file 3: Tables S2a-b). We selected a 10-year exposure lag period because in most instances it had the lowest AIC value.

We observed a monotonic exposure-response relationship for leukemias and the tertile categorization of cumulative exposure to VC and PCE with HRs of 1.01 and 1.00 in the middle exposure category, and HRs of 1.72 (95% CI: 0.33, 8.83) and 1.82 (95% CI: 0.36, 9.32) at the high category exposure level, respectively (Table 5a). A monotonic exposure-response relationship was also found for leukemias and the tertile categorization of average exposure to VC with HRs of 1.64 (95% CI: 0.31, 8.73) and 1.95 (95% CI: 0.37, 10.43) in the middle and high exposure level. Nine of the 12 leukemia deaths had cumulative exposures to each contaminant above the median. Splines for leukemias and cumulative exposures to PCE and VC indicated a steady rise in HRs to a maximum of about 2.2 to 2.3 at the 85^th^ percentile of cumulative exposure and thereafter declining to HRs of about 1.6 (Additional file 4: Figures S1a-b). This decline in the HRs could be due to exposure misclassification bias [22]. The beta coefficients for untransformed cumulative exposure were positive, but the log base 10 beta coefficients were negative (Table 5a). The untransformed and transformed cumulative exposure models had similar AIC values. Of the 6 ANLL deaths, 4 had cumulative exposures above the median for each contaminant.

**Table 5:** Hazard ratios (95% CI) for tertiles of maximum cumulative exposure and coefficients (95% CI) for continuous cumulative exposure (μg/L-year)

**a. Leukemias (N = 12)**				
Contaminant	Medium Exposure	High Exposure	Cumulative Exposure	Log_10_ Cumulative Exposure
PCE	1.00 (0.14, 7.39) N = 2	1.82 (0.36, 9.32) N = 8	0.0010 (−0.0080, 0.0101) p = .82	−0.0498 (−0.7053, 0.6056) p = .88
TCE	0.94 (0.13, 6.97) N = 2	1.65 (0.32, 8.49) N = 8	<0.00001 (−0.0004, 0.0004) p = .84	−0.1712 (−0.6390, 0.2966) p = .47
Vinyl Chloride	1.01 (0.14, 7.45) N = 2	1.72 (0.33, 8.83) N = 8	0.0008 (−0.0051, 0.0067) p = .80	−0.0982 (−0.7363, 0.5398) p = .76
Benzene	0.36 (0.04, 3.52) N = 1	1.25 (0.31, 5.10) N = 8	0.0043 (−0.0206, 0.0292) p = .73	−0.1221 (−0.9360, 0.6918) p = .77
TVOC	0.94 (0.13, 6.97) N = 2	1.68 (0.33, 8.67) N = 8	<0.00001 (−0.0002, 0.0003) p = .83	−0.2334 (−0.7150, 0.2483) p = .34
**b. Prostate Cancer (N = 10)**				
Contaminant	Medium Exposure	High Exposure	Cumulative Exposure	Log_10_ Cumulative Exposure
PCE	3.46 (0.38, 31.65) N = 4	2.08 (0.23, 18.91) N = 5	0.0039 (−0.0059, 0.0137) p = .44	0.3618 (−0.4945, 1.2181) p = .41
TCE	2.55 (0.26, 25.15) N = 3	2.39 (0.27, 21.14) N = 6	0.0002 (−0.0002, 0.0006) p = .37	0.4394 (−0.4270, 1.3058) p = .32
Vinyl Chloride	3.54 (0.39, 32.37) N = 4	2.00 (0.22, 18.21) N = 5	0.0023 (−0.0042, 0.0088) p = .49	0.3317 (−0.5040, 1.1674) p = .44
Benzene	1.60 (0.26, 9.79) N = 3	1.13 (0.21, 6.19) N = 5	0.0083 (−0.0188, 0.0354) p = .55	0.2962 (−0.6663, 1.2587) p = .55
TVOC	2.65 (0.27, 26.15) N = 3	2.47 (0.28, 21.82) N = 6	0.0001 (−0.0001, 0.0003) p = .39	0.4298 (−0.4438, 1.3034) p = .33

Exposure lagged 10 years. Adjusted by sex, race, occupation (blue collar vs white collar) and education. Selected causes of death. Camp Lejeune cohort (N = 4,647). Reference group consists of Camp Lejeune civilian employees in the lowest tertile level of maximum cumulative exposure.

All kidney cancer deaths (n = 7) among the Camp Lejeune cohort had cumulative exposures above the median for PCE, TCE, and VC. Only 1 kidney cancer was below the median for cumulative exposure to TVOC and two were below the median for benzene. Only 1 kidney cancer was below the median average exposure to each of the contaminants. Because of the small numbers and high cumulative and average exposures of kidney cancers, categorical analyses resulted in infinite HRs for some of the contaminants (Table 6a). The AIC values for the untransformed and transformed cumulative exposure models were similar and the beta coefficients were positive.

**Table 6:** Hazard ratios (95% CI) for categorized (<median (ref.), ≥median) maximum cumulative exposure and coefficients (95% CI) for continuous cumulative exposure (μg/L-year)

**a. Kidney Cancer (N = 7)**			
Contaminant	≥ Median Exposure	Cumulative Exposure	Log_10_ Cumulative Exposure
Benzene	1.82 (0.34, 9.78) N = 5	0.0240 (−0.0080, 0.0559) p = .14	1.3595 (−0.3324, 3.0515) p = .11
TVOC	4.44 (0.52, 38.19) N = 6	0.0002 (−0.0001, 0.0006) p = .13	1.3626 (−0.4550, 3.1801) p = .14
PCE	Inf. N = 7	0.0100 (−0.0019, 0.0219) p = .10	1.4753 (−0.2983, 3.2489) p = .10
TCE	Inf. N = 7	0.0004 (−0.0001, 0.0009) p = .12	1.3551 (−0.4666, 3.1768) p = .14
Vinyl Chloride	Inf N = 7	0.0063 (−0.0015, 0.0141) p = .11	1.4370 (−0.3327, 3.2066) p = .11
**b. Parkinson’s Disease (N = 5)**			
Contaminant	≥ Median Exposure	Cumulative Exposure	Log_10_ Cumulative Exposure
PCE	2.68 (0.22, 33.28) N = 4	0.0199 (0.0005, 0.0393) p = .04	1.9718 (−0.8134, 4.7569) p = .16
TCE	2.51 (0.21, 30.76) N = 4	0.0009 (0.0001, 0.0017) p = .04	2.6244 (−0.7668, 6.0156) p = .13
Vinyl Chloride	2.81 (0.23, 34.11) N = 4	0.0129 (0.0005, 0.0253) p = .04	2.0982 (−0.7936, 4.9900) p = .15
Benzene	2.52 (0.20, 31.59) N = 4	0.0490 (0.0008, 0.0971) p = .05	2.0910 (−0.7578, 4.9398) p = .15
TVOC	2.52 (0.21, 30.83) N = 4	0.0005 (<0.0001, 0.0011) p = .04	2.6729 (−0.7448, 6.0905) p = .12

Exposure lagged 10 years. Adjusted by sex, race, occupation (blue collar vs white collar) and education. Selected causes of death. Camp Lejeune cohort (N = 4,647).

Three of the four esophageal cancer deaths had cumulative exposures above the median for each of the contaminants. HRs for the dichotomous cumulative exposure variables (<median, ≥median) for each of the contaminants were above 2.1 with very wide confidence intervals (Additional file 3: Table S2b).

Although no association was observed for cumulative exposure and multiple myeloma, a monotonic relationship was observed for the tertile categorization of average exposure to benzene with HRs of 1.39 (95% CI: 0.12, 15.65) and 3.15 (95% CI: 0.32, 30.82) in the middle and high exposure level, respectively. The tertile categorization of average exposure could not be evaluated for the other contaminants due to small numbers. Four of the six multiple myeloma deaths had higher than the median average exposure for TCE, VC and TVOC with HRs > 1.8 and very wide confidence intervals for the dichotomous average exposure variables.

No other diseases of primary interest were associated with cumulative or average exposures to the contaminants. Among the diseases of secondary interest, four of the five cases of Parkinson’s disease were above the median cumulative exposure for each of the contaminants. Reflecting this fact, the HRs for the dichotomous cumulative exposure variables were >2.50 (Table 6b). The majority of the cases were also above the median average exposure for each of the contaminants. The AIC values for the untransformed and transformed cumulative exposure models were similar, and the beta coefficients were positive.

Of the ten cases of prostate cancer, eight were above the median cumulative exposure for TCE, PCE, and benzene and seven were above the median for VC, and TVOC. The exposure-response relationships based on the tertiles of cumulative exposures were not monotonic, but the HRs were ≥2.00 in the middle and high exposure categories for PCE, TCE, VC and TVOC (Table 5b). Seven cases were also above the median average exposure for TCE and TVOC. The coefficients for the untransformed and log base 10 transformed cumulative exposure variables were positive and the AIC values were similar for these models.

Of the four cases of rectal cancer, all were above the median cumulative exposure for VC, and three out of four were above the median cumulative exposure for the other contaminants. The HRs for the dichotomous cumulative exposure variables were ≥1.75 for each of the contaminants but could not be calculated for VC or PCE (Additional file 3: Table S2b). All of the rectal cancer cases were also above the median average exposure for each of the contaminants.

None of the other diseases of secondary interest were associated with cumulative or average exposure to the contaminants.

Of the smoking-related diseases not known to be associated with solvent exposure, stomach cancer had elevated HRs for the benzene and vinyl chloride dichotomous cumulative exposure variables but not for the other contaminants. The HRs for the cumulative exposures and COPD and cardiovascular disease were less than 1.0 (Additional file 3: Tables S2 a-b).

## Discussion

Diseases of primary interest that were elevated in the Camp Lejeune cohort compared to Camp Pendleton were kidney cancer and the hematopoietic cancers, leukemias and multiple myeloma.

In addition, several of the diseases of secondary interest were also elevated in the Camp Lejeune cohort compared to Camp Pendleton including cancers of the rectum, lung, breast, prostate and oral; Parkinson’s disease and kidney diseases. Confidence intervals were wide due to small numbers of individual causes of death. In analyses internal to the Camp Lejeune cohort, we observed monotonic trends between cumulative exposures to VC and PCE and leukemias. Most or all of the deaths from cancers of the kidney, esophagus, rectum, and prostate, and Parkinson’s disease had cumulative exposures above the median for each of the contaminants and TVOC. Although multiple myeloma was not associated with cumulative exposure, a monotonic exposure-response relationship was observed for average exposure to benzene, and most of the deaths had average exposures above the median for each of the contaminants and TVOC.

There was some consistency between the findings in this study and the findings in a previous mortality study of Marines and Navy personnel at Camp Lejeune [31]. For example, in the previous study, elevated risks were found for kidney cancer, multiple myeloma, leukemia, rectal cancer, lung cancer and prostate cancer when the Camp Lejeune cohort was compared to the Camp Pendleton cohort. These cancers were also elevated in the current study. In both studies, risks were not elevated for bladder cancer, non-Hodgkin lymphoma, colon cancer, and brain cancer. However, the two studies differed on some cancers. For example, cancers of the liver, esophagus, soft tissue, and pancreas were elevated in the previous study but not the current study. In the current study, cancers of the breast and oral cavity were elevated but not in the previous study. Any conclusions concerning the consistency of the findings in the two studies should be tentative because most of the members of the cohorts in these studies were alive at the end of follow-up.

Studies conducted at Cape Cod, MA found associations between PCE contamination and the incidence of several cancers: lung, bladder, rectal, leukemia, and female breast cancer [10-12]. In the comparison between Camp Lejeune and Camp Pendleton, we also observed elevated HRs for lung cancer, rectal cancer, leukemia and breast cancer but not for bladder cancer. In the New Jersey studies, associations were observed for the incidence of specific subgroupings of leukemia and NHL [9]. We did observe elevations in leukemias, but not NHL, in the current study.

When comparing results across these drinking water studies, it must be kept in mind that the exposure situations were very different. New Jersey and Cape Cod populations were exposed to the contaminants for a much longer time than most of the Camp Lejeune cohort and were primarily exposed at their residences rather than their workplaces. Second, the levels and mixtures of contaminants differed among the studies. At Cape Cod, the only contaminant was PCE, and some of the detected levels of PCE in the Cape Cod drinking water were much higher than those detected or estimated at Camp Lejeune. Similar to Camp Lejeune, some of the towns in the New Jersey study had mixtures of TCE, PCE and other contaminants. However, the maximum detected level of TCE in the Hadnot Point drinking water was considerably higher than the maximum levels detected in the drinking water of the New Jersey towns.

The Camp Pendleton cohort appeared to be an appropriate reference population for the Camp Lejeune cohort because the two bases had somewhat similar demographic and occupational characteristics and the healthy worker effect would be similar in both cohorts. Confounding due to unmeasured risk factors would likely be minimal because of the similarities between the two cohorts. The key difference between the cohorts was the drinking water contamination at Camp Lejeune [16].

The strengths of the study included the small percentage of lost to follow-up and a rigorous reconstruction of historical levels of contamination in the Hadnot Point water system. An additional strength was the inclusion of the Camp Pendleton cohort.

One serious limitation of the study was the small numbers of most causes of death which resulted in wide confidence intervals for the measures of effect. Moreover, because of small numbers, it was not possible to evaluate exposure-response relationships for many of the causes of death within the Camp Lejeune cohort. There were small numbers because of the small size of the cohorts, the fact that a majority were under the age of 65 and only 14% had died by the end of the study, and the healthy worker effect bias. Many of the diseases of interest have relatively long survival rates (e.g., cancers of the kidney, bladder, colon, rectal, breast, prostate, soft tissue and non-Hodgkin lymphoma, and Parkinson’s disease) and would require long-term follow-up of the Camp Lejeune cohort to fully evaluate the health impacts of the drinking water exposures. In addition, some cancers of the digestive system and oral cavity/pharynx appear to be underreported on death certificates compared to cancer registry data [32]. There is also evidence that Parkinson’s disease is underreported on death certificates to a higher extent in the southern U.S. than in other areas of the U.S. [33].

Another serious limitation of the study was exposure misclassification bias. There were several sources of exposure misclassification. For example, due to a lack of information on workplace locations, we assumed that all the Camp Lejeune workers were located, or spent considerable time during the work day, at the mainside area of the base served by the Hadnot Point treatment plant. Although this assumption was true for most workers, undoubtedly some did not work in the mainside area.

In addition, we lacked information on water usage of the Camp Lejeune workers. Workers likely varied in their use of drinking water during the workday. Some workers in the mainside area of the base may have been unexposed because they did not use the drinking water for any purpose during the workday.

The exposure misclassification bias was likely considerable but non-differential, i.e., the errors in assigning exposures were likely to be unrelated to disease status. Non-differential exposure misclassification could bias the HRs comparing Camp Lejeune to Camp Pendleton towards the null value of 1.00, resulting in underestimates of the true effect of the exposures [26]. In the analyses of cumulative exposures internal to the Camp Lejeune cohort, such bias could distort exposure-response relationships, for example producing a curve that plateaus or tails off at higher levels of cumulative exposure [22].

Another limitation was the lack of information on smoking and other risk factors such as occupational exposures prior to or after employment at Camp Lejeune or Camp Pendleton. Such risk factors, if associated with exposure status, could act as confounders, biasing the HR towards or away from the null value of 1.00 and distorting exposure-response relationships. Camp Lejeune and Camp Pendleton workers had similar demographics and occupations so it is unlikely that confounding would be a major source of bias in the comparisons between the two bases. It is also unlikely that unmeasured risk factors would be associated with cumulative exposures in the analyses that were conducted internal to the Camp Lejeune cohort.

We evaluated smoking-related diseases that were not known to be associated with solvent exposure to get some idea of the extent of the possible confounding effects of smoking. We observed a slight elevation for COPD in the Camp Lejeune cohort compared to the Camp Pendleton cohort. Based on this finding, the confounding effect of smoking on the HRs comparing Camp Lejeune and Camp Pendleton would be less than 18% which is in the range of what other occupational health studies have observed for the confounding effects of smoking [34]. In the analyses internal to Camp Lejeune, the smoking-related diseases were for the most part negatively associated with cumulative exposure.

Another possible confounder is alcohol consumption. Kidney cancer and the hematopoietic cancers are not known to be associated with alcohol consumption. A recent study also indicated that Parkinson’s disease is unrelated to alcohol consumption [35]. On the other hand, several of the diseases that were elevated in the Camp Lejeune cohort compared to the Camp Pendleton cohort have been associated with alcohol consumption: cancers of the oral cavity, breast, and rectum. Other diseases that have been associated with alcohol consumption were not elevated in the Camp Lejeune cohort compared to the Camp Pendleton cohort: cancers of the liver, esophagus, and colon, cardiovascular diseases and liver diseases. Therefore it does not appear that alcohol was a confounder for the comparisons between Camp Lejeune and Camp Pendleton. Within the Camp Lejeune cohort, cumulative exposures were related to esophageal and rectal cancers but not for other alcohol-related cancers or diseases. Therefore, it does not appear that alcohol was a confounder for these comparisons internal to the Camp Lejeune cohort.

## Conclusion

The study found elevated HRs in the Camp Lejeune cohort for several causes of death, including kidney cancer, leukemia, multiple myeloma, rectal cancer, and Parkinson’s disease. Because only 14% of the Camp Lejeune cohort had died by the end of the study, the number of cause-specific deaths was small resulting in wide confidence intervals. Additional follow-up would be necessary to comprehensively assess effects of drinking water exposures at the base.

## Abbreviations

ATSDR: Agency for toxic substances and disease registry; AIC: Akaike’s information criterion; ALS: Amyotrophic lateral sclerosis; COPD: Chronic obstructive pulmonary disease; CI: Confidence interval; DMF: Death master file; DMDC: Defense manpower data center; HP: Hadnot point; HR: Hazard ratio; LTAS: Life table analysis system; MCL: Maximum contaminant level; μg/L: Micrograms per liter; NDI: National death index; NHL: Non-Hodgkin lymphoma; NTP: National toxicology program; ORES: Office of research, evaluation and statistics; ppm: Parts per million; RCS: Restricted cubic spline functions; SSN: Social security number; SSA: Social security administration; SMR: Standardized mortality ratio; TVOC: Total amount of the contaminants; TCE: Trichloroethylene; PCE: Tetrachloroethylene or perchloroethylene; USMC: United States Marine Corps; EPA: United states environmental protection agency.

## Competing interests

All authors declare they have no actual or potential competing financial interest.

## Authors’ contributions

FJB participated in the study design, data collection, analysis and interpretation of data, and drafted the manuscript. PZR participated in the study design, data collection, interpretation of data, and helped draft the manuscript. MM conducted the water modeling. TCL assisted with analysis and interpretation of data. All authors read and approved the final manuscript.

## Supplementary Material

Additional file 1 Camp Lejeune vs Camp Pendleton stratified by sex, race, and occupation. Click here for file

Additional file 2 **Cumulative Exposures (untransformed and log base 10 transformed), 10 year lag, adjusted.** Camp Lejeune Cohort (N = 4,647) (Causes of death with N ≥5). Click here for file

Additional file 3 **Adjusted Hazard ratios for categorizations of Cumulative exposures, 10 year lag.** (Reference group has no/low cumulative exposure). Camp Lejeune cohort (N = 4,647). Click here for file

Additional file 4 1 Spline of leukemias and cumulative exposure to PCE and vinyl chloride. Click here for file
