# Supplementary material for: Mortality study of civilian employees exposed to contaminated drinking water at USMC Base Camp Lejeune: a retrospective cohort study
Source: Environ Health. 2014 Aug 13;13:68. doi: 10.1186/1476-069X-13-68 (PMC4237831; doi:10.1186/1476-069X-13-68)
Supplement: Additional file 1 — Camp Lejeune vs Camp Pendleton stratified by sex, race, and occupation. [file 1476-069X-13-68-S1.doc]

**Table S3a: Camp Lejeune vs Camp Pendleton stratified by sex**.

| Gender: | Males | | | Females | | |
| --- | --- | --- | --- | --- | --- | --- |
| **Outcome** | **Hazard**  **Ratio** | **Lower CL** | **Upper CL** | **Hazard**  **Ratio** | **Lower CL** | **Upper CL** |
| All Cancers | 1.17 | 0.91 | 1.50 | 0.99 | 0.72 | 1.36 |
| **Diseases of Primary Interest**: | | | | | | |
| Kidney Cancer | 1.90 | 0.48 | 7.58 | 1.54 | 0.13 | 17.98 |
| Hematopoietic Cancers | 2.60 | 1.17 | 5.76 | 0.44 | 0.13 | 1.41 |
| Multiple Myeloma | 3.01 | 0.59 | 15.28 | ̶ |  |  |
| Leukemia | 3.36 | 1.09 | 10.33 | 0.29 | 0.03 | 2.55 |
| Non Hodgkin Lymphoma | 1.28 | 0.25 | 6.71 | 0.65 | 0.12 | 3.55 |
|  |  |  |  |  |  |  |
| **Diseases of Secondary Interest**: | | | | | | |
| Pancreatic Cancer | 0.75 | 0.27 | 2.03 | 0.42 | 0.11 | 1.59 |
| Colorectal Cancer | 1.20 | 0.52 | 2.78 | 0.83 | 0.16 | 4.29 |
| Colon Cancer | 1.15 | 0.43 | 3.06 | 0.52 | 0.08 | 3.29 |
| Brain Cancer | 0.43 | 0.08 | 2.23 | 1.23 | 0.22 | 6.78 |
| Lung Cancer | 1.17 | 0.79 | 1.75 | 1.53 | 0.80 | 2.93 |
| Kidney Diseases | 0.98 | 0.22 | 4.34 | 1.83 | 0.27 | 12.46 |
| Liver Diseases | 0.76 | 0.24 | 2.42 | 1.06 | 0.20 | 5.65 |
| Parkinson’s Disease | 2.25 | 0.47 | 10.70 | 4.66 | 0.29 | 75.00 |
|  |  |  |  |  |  |  |
| **Smoking-related Diseases (not known to be related to solvent exposure):** | | | | | | |
| COPD | 1.51 | 0.88 | 2.60 | 0.83 | 0.37 | 1.87 |
| Cardiovascular Disease | 0.98 | 0.78 | 1.22 | 0.90 | 0.61 | 1.31 |

Adjusted for race, occupation (blue collar/white collar), and education; exposure lagged 10 years.

Excluded because cause of death <5 in the Camp Lejeune cohort: bladder cancer, liver cancer, esophageal cancer, Hodgkin lymphoma, rectal cancer, soft tissue cancers, laryngeal cancer, oral cancers, ALS, MS, and stomach cancer.

Camp Lejeune: Males = 1,991 Females = 2,656

Camp Pendleton: Males = 2,314 Females = 2,376

**Table S3b: Camp Lejeune vs Camp Pendleton stratified by race*.**

| **Outcome** | **Hazard**  **Ratio** | **Lower CL** | **Upper CL** |
| --- | --- | --- | --- |
| All Cancers | 1.08 | 0.88 | 1.33 |
| **Diseases of Primary Interest**: | | | |
| Kidney Cancer | 1.64 | 0.46 | 5.86 |
| Hematopoietic Cancers | 1.39 | 0.72 | 2.68 |
| Multiple Myeloma | 2.32 | 0.42 | 12.92 |
| Leukemia | 1.55 | 0.63 | 3.85 |
| Non Hodgkin Lymphoma | 0.78 | 0.22 | 2.73 |
|  |  |  |  |
| **Diseases of Secondary Interest**: | | | |
| Pancreatic Cancer | 0.56 | 0.24 | 1.31 |
| Colorectal Cancer | 1.10 | 0.49 | 2.47 |
| Colon Cancer | 0.92 | 0.35 | 2.46 |
| Brain Cancer | 0.65 | 0.21 | 2.04 |
| Lung Cancer | 1.24 | 0.87 | 1.76 |
| Kidney Diseases | 0.92 | 0.26 | 3.34 |
| Liver Diseases | 0.90 | 0.32 | 2.48 |
| Parkinson | 1.71 | 0.37 | 7.90 |
|  |  |  |  |
| **Smoking-related Diseases (not known to be related to solvent exposure):** | | | |
| COPD | 1.18 | 0.74 | 1.88 |
| Cardiovascular Disease | 0.88 | 0.71 | 1.09 |

* These analyses are for those of “white” race only. The numbers of African American civilian workers and workers of other races/ethnicities were too small to evaluate separately.

Adjusted for sex, occupation (blue collar/white collar) and education

Excluded because cause of death <5 in the Camp Lejeune cohort: bladder cancer, liver cancer, esophageal cancer, Hodgkin lymphoma, rectal cancer, soft tissue cancers, laryngeal cancer, oral cancers, ALS, MS, and stomach cancer.

Camp Lejeune: white = 3,820 African American = 717 Other = 110

Camp Pendleton: white = 3,694 African American = 421 Other = 575

**Table S3c: Camp Lejeune vs Camp Pendleton stratified by occupation#.**

| Occupation: | White Collar | | | Blue Collar | | |
| --- | --- | --- | --- | --- | --- | --- |
| **Outcome** | **Hazard**  **Ratio** | **Lower CL** | **Upper CL** | **Hazard**  **Ratio** | **Lower CL** | **Upper CL** |
| All Cancers | 1.04 | 0.80 | 1.36 | 1.20 | 0.90 | 1.61 |
| **Diseases of Primary Interest**: | | | | | | |
| Kidney Cancer | 1.50 | 0.29 | 7.88 | 2.01 | 0.36 | 11.21 |
| Hematopoietic Cancers | 0.92 | 0.35 | 2.44 | 2.14 | 0.93 | 4.91 |
| Multiple Myeloma | 3.20 | 0.22 | 46.40 | 1.79 | 0.32 | 9.93 |
| Leukemia | 0.49 | 0.10 | 2.48 | 3.85 | 1.12 | 13.30 |
| Non Hodgkin Lymphoma | 0.84 | 0.14 | 4.86 | 0.89 | 0.19 | 4.23 |
|  |  |  |  |  |  |  |
| **Diseases of Secondary Interest**: | | | | | | |
| Pancreatic Cancer | 0.76 | 0.29 | 1.99 | 0.32 | 0.07 | 1.47 |
| Colorectal Cancer | 1.26 | 0.41 | 3.89 | 1.11 | 0.41 | 2.98 |
| Colon Cancer | 1.32 | 0.38 | 4.51 | 0.84 | 0.25 | 2.78 |
| Brain Cancer | 2.01 | 0.45 | 8.98 | ̶ |  |  |
| Lung Cancer | 1.40 | 0.85 | 2.30 | 1.17 | 0.74 | 1.85 |
| Kidney Diseases | 1.33 | 0.18 | 9.66 | 1.27 | 0.31 | 5.17 |
| Liver Diseases | 1.15 | 0.19 | 7.09 | 0.71 | 0.23 | 2.24 |
| Parkinson’s Disease | 1.25 | 0.13 | 12.34 | 5.73 | 0.60 | 54.27 |
|  |  |  |  |  |  |  |
| **Smoking-related Diseases (not known to be related to solvent exposure):** | | | | | | |
| COPD | 0.95 | 0.48 | 1.90 | 1.46 | 0.81 | 2.65 |
| Cardiovascular Disease | 0.94 | 0.70 | 1.26 | 0.99 | 0.77 | 1.28 |

# Occupation was stratified as blue collar and white collar.

Adjusted for race, sex, and education

Excluded because cause of death <5 in the Camp Lejeune cohort: bladder cancer, liver cancer, esophageal cancer, Hodgkin lymphoma, rectal cancer, soft tissue cancers, laryngeal cancer, oral cancers, ALS, MS, and stomach cancer.

Camp Lejeune: White Collar = 3,240 Blue Collar = 1,407

Camp Pendleton: White Collar = 3,041 Blue Collar = 1,649
