# Supplementary material for: Mortality study of civilian employees exposed to contaminated drinking water at USMC Base Camp Lejeune: a retrospective cohort study
Source: Environ Health. 2014 Aug 13;13:68. doi: 10.1186/1476-069X-13-68 (PMC4237831; doi:10.1186/1476-069X-13-68)
Supplement: Additional file 2 — Cumulative Exposures (untransformed and log base 10 transformed), 10 year lag, adjusted. Camp Lejeune Cohort (N = 4,647) (Causes of death with N ≥5). [file 1476-069X-13-68-S2.docx]

**Table S1a: Cumulative Exposures (untransformed), 10 year lag, adjusted.**

**Camp Lejeune Cohort (N=4,647)** (Causes of death with N ≥5)

| **Underlying Cause of Death** | **Cumulative Exposure** | **Beta coefficient**  **(µg/L-year)** | **S.E.** | **P-value** | **95% LCL** | **95% UCL** |
| --- | --- | --- | --- | --- | --- | --- |
| All Cancers | PCE | -0.0012 | 0.0012 | 0.31 | -0.0035 | 0.0011 |
| **Diseases of Primary Interest**: |  |  |  |  |  |  |
| Kidney Cancer | PCE | 0.0100 | 0.0061 | 0.10 | -0.0019 | 0.0219 |
| Hematopoietic Cancers | PCE | 0.0004 | 0.0033 | 0.90 | -0.0061 | 0.0069 |
| Non-Hodgkin Lymphoma | PCE | -0.0084 | 0.0088 | 0.34 | -0.0256 | 0.0089 |
| Leukemias | PCE | 0.0010 | 0.0046 | 0.82 | -0.0080 | 0.0101 |
| Multiple Myeloma | PCE | 0.0038 | 0.0063 | 0.54 | -0.0085 | 0.0162 |
| **Diseases of Secondary Interest**: |  |  |  |  |  |  |
| Colorectal Cancer | PCE | -0.0023 | 0.0046 | 0.62 | -0.0113 | 0.0068 |
| Colon Cancer | PCE | -0.0092 | 0.0068 | 0.18 | -0.0226 | 0.0041 |
| Pancreatic Cancer | PCE | -0.0027 | 0.0055 | 0.63 | -0.0134 | 0.0081 |
| Lung Cancer | PCE | -0.0027 | 0.0020 | 0.17 | -0.0065 | 0.0012 |
| Brain Cancer | PCE | -0.0156 | 0.0149 | 0.29 | -0.0449 | 0.0137 |
| Female Breast Cancer | PCE | -0.0033 | 0.0055 | 0.56 | -0.0141 | 0.0075 |
| Prostate Cancer | PCE | 0.0039 | 0.0050 | 0.44 | -0.0059 | 0.0137 |
| Liver Diseases | PCE | 0.0006 | 0.0059 | 0.92 | -0.0111 | 0.0123 |
| Kidney Diseases | PCE | -0.0033 | 0.0064 | 0.60 | -0.0160 | 0.0093 |
| Parkinson’s Disease | PCE | 0.0199 | 0.0099 | 0.04 | 0.0005 | 0.0393 |
| **Smoking-related Diseases (not known to be related to solvent exposure):** | | | | | | |
| Cardiovascular Diseases | PCE | -0.0010 | 0.0012 | 0.40 | -0.0033 | 0.0013 |
| COPD | PCE | -0.0036 | 0.0027 | 0.19 | -0.0089 | 0.0017 |
| All Cancers | TCE | -0.0001 | 0.0001 | 0.31 | -0.0002 | 0.0001 |
| **Diseases of Primary Interest**: |  |  |  |  |  |  |
| Kidney Cancer | TCE | 0.0004 | 0.0003 | 0.12 | -0.0001 | 0.0009 |
| Hematopoietic Cancers | TCE | 0.0000 | 0.0001 | 0.85 | -0.0003 | 0.0003 |
| Non-Hodgkin Lymphoma | TCE | -0.0003 | 0.0004 | 0.37 | -0.0011 | 0.0004 |
| Leukemias | TCE | 0.0000 | 0.0002 | 0.84 | -0.0004 | 0.0004 |
| Multiple Myeloma | TCE | 0.0002 | 0.0003 | 0.49 | -0.0003 | 0.0007 |
| **Diseases of Secondary Interest**: |  |  |  |  |  |  |
| Colorectal Cancer | TCE | -0.0001 | 0.0002 | 0.58 | -0.0005 | 0.0003 |
| Colon Cancer | TCE | -0.0004 | 0.0003 | 0.17 | -0.0010 | 0.0002 |
| Pancreatic Cancer | TCE | -0.0001 | 0.0002 | 0.63 | -0.0006 | 0.0004 |
| Lung Cancer | TCE | -0.0001 | 0.0001 | 0.17 | -0.0003 | 0.0001 |
| Brain Cancer | TCE | -0.0004 | 0.0005 | 0.41 | -0.0015 | 0.0006 |
| Female Breast Cancer | TCE | -0.0002 | 0.0003 | 0.47 | -0.0008 | 0.0004 |
| Prostate Cancer | TCE | 0.0002 | 0.0002 | 0.37 | -0.0002 | 0.0006 |
| Liver Diseases | TCE | 0.0000 | 0.0003 | 0.91 | -0.0005 | 0.0005 |
| Kidney Diseases | TCE | -0.0001 | 0.0003 | 0.61 | -0.0007 | 0.0004 |
| Parkinson’s Disease | TCE | 0.0009 | 0.0004 | 0.04 | 0.0001 | 0.0017 |
| **Underlying Cause of Death** | **Cumulative Exposure** | **Beta coefficient**  **(µg/L-year)** | **S.E.** | **P-value** | **95% LCL** | **95% UCL** |
| **Smoking-related Diseases (not known to be related to solvent exposure):** | | | | | | |
| Cardiovascular Diseases | TCE | 0.0000 | 0.0001 | 0.42 | -0.0001 | 0.0001 |
| COPD | TCE | -0.0001 | 0.0001 | 0.22 | -0.0004 | 0.0001 |
| All Cancers | VC | -0.0008 | 0.0008 | 0.28 | -0.0023 | 0.0007 |
| **Diseases of Primary Interest**: |  |  |  |  |  |  |
| Kidney Cancer | VC | 0.0063 | 0.0039 | 0.11 | -0.0015 | 0.0141 |
| Hematopoietic Cancers | VC | 0.0002 | 0.0022 | 0.91 | -0.0040 | 0.0045 |
| Non-Hodgkin Lymphoma | VC | -0.0056 | 0.0058 | 0.33 | -0.0171 | 0.0058 |
| Leukemias | VC | 0.0008 | 0.0030 | 0.80 | -0.0051 | 0.0067 |
| Multiple Myeloma | VC | 0.0024 | 0.0041 | 0.56 | -0.0056 | 0.0105 |
| **Diseases of Secondary Interest**: |  |  |  |  |  |  |
| Colorectal Cancer | VC | -0.0015 | 0.0030 | 0.63 | -0.0073 | 0.0044 |
| Colon Cancer | VC | -0.0060 | 0.0044 | 0.17 | -0.0147 | 0.0027 |
| Pancreatic Cancer | VC | -0.0019 | 0.0036 | 0.60 | -0.0089 | 0.0052 |
| Lung Cancer | VC | -0.0018 | 0.0013 | 0.17 | -0.0043 | 0.0008 |
| Brain Cancer | VC | -0.0102 | 0.0097 | 0.29 | -0.0293 | 0.0089 |
| Female Breast Cancer | VC | -0.0015 | 0.0035 | 0.67 | -0.0084 | 0.0054 |
| Prostate Cancer | VC | 0.0023 | 0.0033 | 0.49 | -0.0042 | 0.0088 |
| Liver Diseases | VC | 0.0005 | 0.0038 | 0.91 | -0.0071 | 0.0080 |
| Kidney Diseases | VC | -0.0020 | 0.0042 | 0.64 | -0.0102 | 0.0062 |
| Parkinson’s Disease | VC | 0.0129 | 0.0063 | 0.04 | 0.0005 | 0.0253 |
| **Smoking-related Diseases (not known to be related to solvent exposure):** | | | | | | |
| Cardiovascular Diseases | VC | -0.0006 | 0.0008 | 0.40 | -0.0022 | 0.0009 |
| COPD | VC | -0.0024 | 0.0018 | 0.17 | -0.0059 | 0.0011 |
| All Cancers | Benzene | -0.0039 | 0.0032 | 0.23 | -0.0102 | 0.0025 |
| **Diseases of Primary Interest**: |  |  |  |  |  |  |
| Kidney Cancer | Benzene | 0.0240 | 0.0163 | 0.14 | -0.0080 | 0.0559 |
| Hematopoietic Cancers | Benzene | 0.0005 | 0.0091 | 0.95 | -0.0174 | 0.0185 |
| Non-Hodgkin Lymphoma | Benzene | -0.0246 | 0.0249 | 0.32 | -0.0736 | 0.0243 |
| Leukemias | Benzene | 0.0043 | 0.0127 | 0.73 | -0.0206 | 0.0292 |
| Multiple Myeloma | Benzene | 0.0075 | 0.0173 | 0.66 | -0.0264 | 0.0414 |
| **Diseases of Secondary Interest**: |  |  |  |  |  |  |
| Colorectal Cancer | Benzene | -0.0084 | 0.0128 | 0.51 | -0.0336 | 0.0168 |
| Colon Cancer | Benzene | -0.0245 | 0.0185 | 0.18 | -0.0608 | 0.0117 |
| Pancreatic Cancer | Benzene | -0.0080 | 0.0149 | 0.59 | -0.0373 | 0.0213 |
| Lung Cancer | Benzene | -0.0078 | 0.0054 | 0.15 | -0.0184 | 0.0028 |
| Brain Cancer | Benzene | -0.0525 | 0.0462 | 0.26 | -0.1433 | 0.0383 |
| Female Breast Cancer | Benzene | -0.0046 | 0.0142 | 0.75 | -0.0325 | 0.0233 |
| Prostate Cancer | Benzene | 0.0083 | 0.0138 | 0.55 | -0.0188 | 0.0354 |
| Liver Diseases | Benzene | 0.0005 | 0.0161 | 0.97 | -0.0311 | 0.0322 |
| Kidney Diseases | Benzene | -0.0087 | 0.0177 | 0.62 | -0.0435 | 0.0261 |
| Parkinson’s Disease | Benzene | 0.0490 | 0.0245 | 0.05 | 0.0008 | 0.0971 |
| **Underlying Cause of Death** | **Cumulative Exposure** | **Beta coefficient**  **(µg/L-year)** | **S.E.** | **P-value** | **95% LCL** | **95% UCL** |
| **Smoking-related Diseases (not known to be related to solvent exposure):** | | | | | | |
| Cardiovascular Diseases | Benzene | -0.0030 | 0.0032 | 0.36 | -0.0093 | 0.0034 |
| COPD | Benzene | -0.0110 | 0.0075 | 0.14 | -0.0257 | 0.0037 |
| All Cancers | TVOC | 0.0000 | 0.0000 | 0.30 | -0.0001 | 0.0000 |
| **Diseases of Primary Interest**: |  |  |  |  |  |  |
| Kidney Cancer | TVOC | 0.0002 | 0.0002 | 0.13 | -0.0001 | 0.0006 |
| Hematopoietic Cancers | TVOC | 0.0000 | 0.0001 | 0.85 | -0.0002 | 0.0002 |
| Non-Hodgkin Lymphoma | TVOC | -0.0002 | 0.0002 | 0.37 | -0.0007 | 0.0003 |
| Leukemias | TVOC | 0.0000 | 0.0001 | 0.83 | -0.0002 | 0.0003 |
| Multiple Myeloma | TVOC | 0.0001 | 0.0002 | 0.49 | -0.0002 | 0.0005 |
| **Diseases of Secondary Interest**: |  |  |  |  |  |  |
| Colorectal Cancer | TVOC | -0.0001 | 0.0001 | 0.58 | -0.0003 | 0.0002 |
| Colon Cancer | TVOC | -0.0003 | 0.0002 | 0.17 | -0.0006 | 0.0001 |
| Pancreatic Cancer | TVOC | -0.0001 | 0.0002 | 0.62 | -0.0004 | 0.0002 |
| Lung Cancer | TVOC | -0.0001 | 0.0001 | 0.16 | -0.0002 | 0.0000 |
| Brain Cancer | TVOC | -0.0003 | 0.0003 | 0.42 | -0.0009 | 0.0004 |
| Female Breast Cancer | TVOC | -0.0001 | 0.0002 | 0.52 | -0.0005 | 0.0003 |
| Prostate Cancer | TVOC | 0.0001 | 0.0001 | 0.39 | -0.0001 | 0.0003 |
| Liver Diseases | TVOC | 0.0000 | 0.0002 | 0.90 | -0.0003 | 0.0003 |
| Kidney Diseases | TVOC | -0.0001 | 0.0002 | 0.61 | -0.0004 | 0.0003 |
| Parkinson’s Disease | TVOC | 0.0005 | 0.0003 | 0.04 | 0.0000 | 0.0011 |
| **Smoking-related Diseases (not known to be related to solvent exposure):** | | | | | | |
| Cardiovascular Diseases | TVOC | 0.0000 | 0.0000 | 0.43 | -0.0001 | 0.0000 |
| COPD | TVOC | -0.0001 | 0.0001 | 0.22 | -0.0002 | 0.0001 |

**Table S1b: Cumulative Exposures (log base10 transformed), 10 year lag, adjusted.**

**Camp Lejeune Cohort (N=4,647)** (Causes of death with N ≥5)

| **Underlying Cause of Death** | **Log base 10 Cumulative Exposure** | **Beta coefficient**  **(µg/L-year)** | **S.E.** | **P-value** | **95% LCL** | **95% UCL** |
| --- | --- | --- | --- | --- | --- | --- |
| All Cancers | PCE | -0.1104 | 0.0647 | 0.09 | -0.2376 | 0.0168 |
| **Diseases of Primary Interest**: | | | | | | |
| Kidney Cancer | PCE | 1.4753 | 0.9026 | 0.10 | -0.2983 | 3.2489 |
| Hematopoietic Cancers | PCE | -0.0267 | 0.2214 | 0.90 | -0.4617 | 0.4084 |
| Non-Hodgkin Lymphoma | PCE | -0.3945 | 0.4493 | 0.38 | -1.2774 | 0.4884 |
| Leukemias | PCE | -0.0498 | 0.3336 | 0.88 | -0.7053 | 0.6056 |
| Multiple Myeloma | PCE | 0.1189 | 0.4266 | 0.78 | -0.7193 | 0.9570 |
| **Diseases of Secondary Interest**: | | | | | | |
| Colorectal Cancer | PCE | -0.0995 | 0.2318 | 0.67 | -0.5550 | 0.3560 |
| Colon Cancer | PCE | -0.2885 | 0.2521 | 0.25 | -0.7839 | 0.2070 |
| Pancreatic Cancer | PCE | -0.4689 | 0.2470 | 0.06 | -0.9542 | 0.0164 |
| Lung Cancer | PCE | -0.1171 | 0.1098 | 0.29 | -0.3328 | 0.0987 |
| Brain Cancer | PCE | -0.3113 | 0.3461 | 0.37 | -0.9915 | 0.3688 |
| Female Breast Cancer | PCE | -0.2648 | 0.2139 | 0.22 | -0.6851 | 0.1555 |
| Prostate Cancer | PCE | 0.3618 | 0.4358 | 0.41 | -0.4945 | 1.2181 |
| Liver Diseases | PCE | -0.1316 | 0.3128 | 0.67 | -0.7462 | 0.4831 |
| Kidney Diseases | PCE | -0.3190 | 0.3852 | 0.41 | -1.0759 | 0.4380 |
| Parkinson’s Disease | PCE | 1.9718 | 1.4174 | 0.16 | -0.8134 | 4.7569 |
| **Smoking-related Diseases (not known to be related to solvent exposure):** | | | | | | |
| Cardiovascular Diseases | PCE | -0.1016 | 0.0680 | 0.14 | -0.2353 | 0.0320 |
| COPD | PCE | -0.1309 | 0.1613 | 0.42 | -0.4479 | 0.1861 |
|  |  |  |  |  |  |  |
| All Cancers | TCE | -0.0843 | 0.0464 | 0.07 | -0.1754 | 0.0069 |
| **Diseases of Primary Interest**: | | | | | | |
| Kidney Cancer | TCE | 1.3551 | 0.9271 | 0.14 | -0.4666 | 3.1768 |
| Hematopoietic Cancers | TCE | -0.0141 | 0.1706 | 0.93 | -0.3493 | 0.3212 |
| Non-Hodgkin Lymphoma | TCE | -0.1159 | 0.3670 | 0.75 | -0.8371 | 0.6052 |
| Leukemias | TCE | -0.1712 | 0.2381 | 0.47 | -0.6390 | 0.2966 |
| Multiple Myeloma | TCE | 0.1799 | 0.3598 | 0.62 | -0.5270 | 0.8868 |
| **Diseases of Secondary Interest**: | | | | | | |
| Colorectal Cancer | TCE | 0.0020 | 0.1731 | 0.99 | -0.3382 | 0.3422 |
| Colon Cancer | TCE | -0.0971 | 0.1800 | 0.59 | -0.4508 | 0.2566 |
| Pancreatic Cancer | TCE | -0.3255 | 0.1624 | 0.05 | -0.6447 | -0.0064 |
| Lung Cancer | TCE | -0.0847 | 0.0802 | 0.29 | -0.2424 | 0.0730 |
| Brain Cancer | TCE | -0.1283 | 0.2306 | 0.58 | -0.5814 | 0.3248 |
| Female Breast Cancer | TCE | -0.2393 | 0.1369 | 0.08 | -0.5083 | 0.0297 |
| Prostate Cancer | TCE | 0.4394 | 0.4409 | 0.32 | -0.4270 | 1.3058 |
| Liver Diseases | TCE | -0.0293 | 0.2300 | 0.90 | -0.4812 | 0.4227 |
| Kidney Diseases | TCE | -0.2567 | 0.2769 | 0.35 | -0.8007 | 0.2873 |
| Parkinson’s Disease | TCE | 2.6244 | 1.7258 | 0.13 | -0.7668 | 6.0156 |
| **Underlying Cause of Death** | **Log base 10 Cumulative Exposure** | **Beta coefficient**  **(µg/L-year)** | **S.E.** | **P-value** | **95% LCL** | **95% UCL** |
| **Smoking-related Diseases (not known to be related to solvent exposure):** | | | | | | |
| Cardiovascular Diseases | TCE | -0.0651 | 0.0495 | 0.19 | -0.1624 | 0.0323 |
| COPD | TCE | -0.0298 | 0.1300 | 0.82 | -0.2853 | 0.2256 |
| All Cancers | VC | -0.1069 | 0.0626 | 0.09 | -0.2299 | 0.0162 |
| **Diseases of Primary Interest**: | | | | | | |
| Kidney Cancer | VC | 1.4370 | 0.9006 | 0.11 | -0.3327 | 3.2066 |
| Hematopoietic Cancers | VC | -0.0300 | 0.2174 | 0.89 | -0.4573 | 0.3973 |
| Non-Hodgkin Lymphoma | VC | -0.3217 | 0.4472 | 0.47 | -1.2005 | 0.5571 |
| Leukemias | VC | -0.0982 | 0.3247 | 0.76 | -0.7363 | 0.5398 |
| Multiple Myeloma | VC | 0.1243 | 0.4178 | 0.77 | -0.6966 | 0.9452 |
| **Diseases of Secondary Interest**: | | | | | | |
| Colorectal Cancer | VC | -0.0340 | 0.2281 | 0.88 | -0.4822 | 0.4142 |
| Colon Cancer | VC | -0.1999 | 0.2428 | 0.41 | -0.6771 | 0.2773 |
| Pancreatic Cancer | VC | -0.4437 | 0.2317 | 0.06 | -0.8990 | 0.0116 |
| Lung Cancer | VC | -0.1163 | 0.1066 | 0.28 | -0.3257 | 0.0931 |
| Brain Cancer | VC | -0.2541 | 0.3257 | 0.44 | -0.8942 | 0.3860 |
| Female Breast Cancer | VC | -0.2602 | 0.2016 | 0.20 | -0.6563 | 0.1359 |
| Prostate Cancer | VC | 0.3317 | 0.4253 | 0.44 | -0.5040 | 1.1674 |
| Liver Diseases | VC | -0.1066 | 0.3014 | 0.72 | -0.6989 | 0.4857 |
| Kidney Diseases | VC | -0.3302 | 0.3740 | 0.38 | -1.0652 | 0.4047 |
| Parkinson’s Disease | VC | 2.0982 | 1.4717 | 0.15 | -0.7936 | 4.9900 |
| **Smoking-related Diseases (not known to be related to solvent exposure):** | | | | | | |
| Cardiovascular Diseases | VC | -0.0879 | 0.0661 | 0.18 | -0.2178 | 0.0421 |
| COPD | VC | -0.1136 | 0.1594 | 0.48 | -0.4269 | 0.1997 |
| All Cancers | Benzene | -0.1546 | 0.0816 | 0.06 | -0.3149 | 0.0057 |
| **Diseases of Primary Interest**: | | | | | | |
| Kidney Cancer | Benzene | 1.3595 | 0.8611 | 0.11 | -0.3324 | 3.0515 |
| Hematopoietic Cancers | Benzene | -0.1543 | 0.2667 | 0.56 | -0.6784 | 0.3699 |
| Non-Hodgkin Lymphoma | Benzene | -0.6203 | 0.5608 | 0.27 | -1.7223 | 0.4817 |
| Leukemias | Benzene | -0.1221 | 0.4142 | 0.77 | -0.9360 | 0.6918 |
| Multiple Myeloma | Benzene | -0.0321 | 0.4806 | 0.95 | -0.9765 | 0.9123 |
| **Diseases of Secondary Interest**: | | | | | | |
| Colorectal Cancer | Benzene | -0.0940 | 0.2920 | 0.75 | -0.6678 | 0.4798 |
| Colon Cancer | Benzene | -0.2978 | 0.3161 | 0.35 | -0.9188 | 0.3233 |
| Pancreatic Cancer | Benzene | -0.4719 | 0.3240 | 0.15 | -1.1086 | 0.1649 |
| Lung Cancer | Benzene | -0.2422 | 0.1348 | 0.07 | -0.5071 | 0.0228 |
| Brain Cancer | Benzene | -0.4677 | 0.4350 | 0.28 | -1.3224 | 0.3870 |
| Female Breast Cancer | Benzene | -0.1170 | 0.2987 | 0.70 | -0.7039 | 0.4699 |
| Prostate Cancer | Benzene | 0.2962 | 0.4898 | 0.55 | -0.6663 | 1.2587 |
| Liver Diseases | Benzene | -0.1697 | 0.3850 | 0.66 | -0.9262 | 0.5868 |
| Kidney Diseases | Benzene | -0.5127 | 0.4698 | 0.28 | -1.4359 | 0.4105 |
| Parkinson’s Disease | Benzene | 2.0910 | 1.4498 | 0.15 | -0.7578 | 4.9398 |
| **Underlying Cause of Death** | **Log base 10 Cumulative Exposure** | **Beta coefficient**  **(µg/L-year)** | **S.E.** | **P-value** | **95% LCL** | **95% UCL** |
| **Smoking-related Diseases (not known to be related to solvent exposure):** | | | | | | |
| Cardiovascular Diseases | Benzene | -0.1109 | 0.0861 | 0.20 | -0.2801 | 0.0582 |
| COPD | Benzene | -0.2741 | 0.1972 | 0.16 | -0.6616 | 0.1134 |
|  |  |  |  |  |  |  |
| All Cancers | TVOC | -0.0950 | 0.0473 | 0.04 | -0.1880 | -0.0020 |
| **Diseases of Primary Interest**: | | | | | | |
| Kidney Cancer | TVOC | 1.3626 | 0.9249 | 0.14 | -0.4550 | 3.1801 |
| Hematopoietic Cancers | TVOC | -0.0398 | 0.1720 | 0.82 | -0.3778 | 0.2982 |
| Non-Hodgkin Lymphoma | TVOC | -0.1137 | 0.3767 | 0.76 | -0.8539 | 0.6264 |
| Leukemias | TVOC | -0.2334 | 0.2451 | 0.34 | -0.7150 | 0.2483 |
| Multiple Myeloma | TVOC | 0.1729 | 0.3591 | 0.63 | -0.5328 | 0.8786 |
| **Diseases of Secondary Interest**: | | | | | | |
| Colorectal Cancer | TVOC | -0.0061 | 0.1741 | 0.97 | -0.3481 | 0.3360 |
| Colon Cancer | TVOC | -0.1029 | 0.1798 | 0.57 | -0.4563 | 0.2504 |
| Pancreatic Cancer | TVOC | -0.3284 | 0.1681 | 0.05 | -0.6586 | 0.0018 |
| Lung Cancer | TVOC | -0.1140 | 0.0805 | 0.16 | -0.2721 | 0.0442 |
| Brain Cancer | TVOC | -0.1470 | 0.2278 | 0.52 | -0.5947 | 0.3007 |
| Female Breast Cancer | TVOC | -0.2255 | 0.1467 | 0.12 | -0.5138 | 0.0628 |
| Prostate Cancer | TVOC | 0.4298 | 0.4446 | 0.33 | -0.4438 | 1.3034 |
| Liver Diseases | TVOC | 0.0026 | 0.2375 | 0.99 | -0.4641 | 0.4693 |
| Kidney Diseases | TVOC | -0.2875 | 0.2751 | 0.30 | -0.8281 | 0.2530 |
| Parkinson’s Disease | TVOC | 2.6729 | 1.7393 | 0.12 | -0.7448 | 6.0905 |
| **Smoking-related Diseases (not known to be related to solvent exposure):** | | | | | | |
| Cardiovascular Diseases | TVOC | -0.0616 | 0.0510 | 0.23 | -0.1618 | 0.0386 |
| COPD | TVOC | -0.0515 | 0.1324 | 0.70 | -0.3115 | 0.2086 |

Models were adjusted by sex, race, occupation (blue collar vs white collar) and education level.

TVOC: total contaminants (PCE, TCE, t-1,2-dichloroethylene, VC, and benzene)

VC: vinyl chloride

TCE: trichloroethylene

PCE: perchloroethylene (or tetrachloroethylene)
