# Supplementary material for: Mortality study of civilian employees exposed to contaminated drinking water at USMC Base Camp Lejeune: a retrospective cohort study
Source: Environ Health. 2014 Aug 13;13:68. doi: 10.1186/1476-069X-13-68 (PMC4237831; doi:10.1186/1476-069X-13-68)
Supplement: Additional file 3 — Adjusted Hazard ratios for categorizations of Cumulative exposures, 10 year lag. (Reference group has no/low cumulative exposure). Camp Lejeune cohort (N = 4,647). [file 1476-069X-13-68-S3.docx]

**Table S2a: Adjusted Hazard ratios for tertiles of Cumulative exposures, 10 year lag. (Reference group has no/low cumulative exposure). Camp Lejeune cohort (N=4,647)** (Causes of death with N ≥10)

| Underlying Cause | Contaminant | Medium Exposure | 95% LCL | 95% UCL | P-value | High Exposure | 95% LCL | 95% UCL | P-value |
| --- | --- | --- | --- | --- | --- | --- | --- | --- | --- |
| All Cancers | TVOC | 0.79 | 0.56 | 1.12 | 0.19 | 0.85 | 0.62 | 1.18 | 0.34 |
| **Diseases of Primary Interest**: | | | | | | | | | |
| Hematopoietic Cancers | TVOC | 0.64 | 0.20 | 2.08 | 0.46 | 0.81 | 0.30 | 2.18 | 0.68 |
| Leukemia | TVOC | 0.94 | 0.13 | 6.97 | 0.95 | 1.68 | 0.33 | 8.67 | 0.53 |
| **Diseases of Secondary Interest**: | | | | | | | | | |
| Lung Cancer | TVOC | 0.92 | 0.52 | 1.63 | 0.79 | 0.77 | 0.45 | 1.33 | 0.35 |
| Pancreatic Cancer | TVOC | 0.12 | 0.01 | 0.97 | 0.05 | 0.43 | 0.12 | 1.50 | 0.18 |
| Colorectal Cancer | TVOC | 1.21 | 0.34 | 4.31 | 0.76 | 0.99 | 0.28 | 3.47 | 0.99 |
| Colon Cancer | TVOC | 1.03 | 0.27 | 3.96 | 0.97 | 0.53 | 0.12 | 2.41 | 0.41 |
| Breast Cancer | TVOC | 1.17 | 0.44 | 3.14 | 0.75 | 0.84 | 0.24 | 2.91 | 0.78 |
| Prostate Cancer | TVOC | 2.65 | 0.27 | 26.15 | 0.40 | 2.47 | 0.28 | 21.82 | 0.42 |
| **Smoking-related Diseases (not known to be related to solvent exposure):** | | | | | | | | | |
| COPD | TVOC | 0.80 | 0.35 | 1.83 | 0.59 | 0.68 | 0.32 | 1.44 | 0.31 |
| Cardiovascular Disease | TVOC | 0.71 | 0.49 | 1.03 | 0.07 | 0.79 | 0.56 | 1.10 | 0.16 |
| All Cancers | benzene | 0.84 | 0.60 | 1.18 | 0.31 | 0.87 | 0.63 | 1.20 | 0.39 |
| **Diseases of Primary Interest**: | | | | | | | | | |
| Hematopoietic Cancers | benzene | 0.35 | 0.09 | 1.30 | 0.12 | 0.70 | 0.28 | 1.76 | 0.45 |
| Leukemia | benzene | 0.36 | 0.04 | 3.52 | 0.38 | 1.25 | 0.31 | 5.10 | 0.76 |
| **Diseases of Secondary Interest**: | | | | | | | | | |
| Lung Cancer | benzene | 0.74 | 0.41 | 1.33 | 0.31 | 0.81 | 0.48 | 1.36 | 0.43 |
| Pancreatic Cancer | benzene | 0.47 | 0.11 | 1.90 | 0.29 | 0.41 | 0.10 | 1.68 | 0.21 |
| Colorectal Cancer | benzene | 1.55 | 0.46 | 5.18 | 0.48 | 0.88 | 0.24 | 3.25 | 0.85 |
| Colon Cancer | benzene | 1.36 | 0.38 | 4.83 | 0.63 | 0.38 | 0.07 | 2.08 | 0.26 |
| Breast Cancer | benzene | 1.69 | 0.62 | 4.60 | 0.31 | 1.09 | 0.30 | 3.92 | 0.90 |
| Prostate Cancer | benzene | 1.60 | 0.26 | 9.79 | 0.61 | 1.13 | 0.21 | 6.19 | 0.89 |
| **Smoking-related Diseases (not known to be related to solvent exposure):** | | | | | | | | | |
| COPD | benzene | 0.47 | 0.20 | 1.09 | 0.08 | 0.46 | 0.22 | 0.94 | 0.03 |
| Cardiovascular Disease | benzene | 0.94 | 0.66 | 1.35 | 0.74 | 0.88 | 0.63 | 1.23 | 0.45 |
| Underlying Cause | Contaminant | Medium Exposure | 95% LCL | 95% UCL | P-value | High Exposure | 95% LCL | 95% UCL | P-value |
| All Cancers | VC | 0.90 | 0.64 | 1.26 | 0.54 | 0.83 | 0.60 | 1.15 | 0.26 |
| **Diseases of Primary Interest**: | | | | | | | | | |
| Hematopoietic Cancers | VC | 0.69 | 0.21 | 2.21 | 0.53 | 0.83 | 0.31 | 2.22 | 0.71 |
| Leukemia | VC | 1.01 | 0.14 | 7.45 | 0.99 | 1.72 | 0.33 | 8.83 | 0.52 |
| **Diseases of Secondary Interest**: | | | | | | | | | |
| Lung Cancer | VC | 0.99 | 0.56 | 1.74 | 0.97 | 0.76 | 0.44 | 1.31 | 0.32 |
| Pancreatic Cancer | VC | 0.42 | 0.10 | 1.71 | 0.23 | 0.38 | 0.09 | 1.55 | 0.18 |
| Colorectal Cancer | VC | 1.20 | 0.34 | 4.27 | 0.77 | 0.99 | 0.28 | 3.49 | 0.99 |
| Colon Cancer | VC | 1.02 | 0.27 | 3.93 | 0.97 | 0.54 | 0.12 | 2.41 | 0.42 |
| Breast Cancer | VC | 1.19 | 0.45 | 3.18 | 0.73 | 0.88 | 0.25 | 3.07 | 0.84 |
| Prostate Cancer | VC | 3.54 | 0.39 | 32.37 | 0.26 | 2.00 | 0.22 | 18.21 | 0.54 |
| **Smoking-related Diseases (not known to be related to solvent exposure):** | | | | | | | | | |
| COPD | VC | 0.64 | 0.28 | 1.45 | 0.28 | 0.54 | 0.26 | 1.14 | 0.11 |
| Cardiovascular Disease | VC | 0.72 | 0.50 | 1.04 | 0.08 | 0.78 | 0.56 | 1.08 | 0.14 |
|  |  |  |  |  |  |  |  |  |  |
| All Cancers | TCE | 0.78 | 0.55 | 1.10 | 0.16 | 0.83 | 0.60 | 1.15 | 0.26 |
| **Diseases of Primary Interest**: | | | | | | | | | |
| Hematopoietic Cancers | TCE | 0.64 | 0.20 | 2.06 | 0.45 | 0.79 | 0.30 | 2.14 | 0.65 |
| Leukemia | TCE | 0.94 | 0.13 | 6.97 | 0.95 | 1.65 | 0.32 | 8.49 | 0.55 |
| **Diseases of Secondary Interest**: | | | | | | | | | |
| Lung Cancer | TCE | 0.91 | 0.52 | 1.62 | 0.76 | 0.75 | 0.44 | 1.30 | 0.31 |
| Pancreatic Cancer | TCE | 0.11 | 0.01 | 0.94 | 0.04 | 0.41 | 0.12 | 1.45 | 0.17 |
| Colorectal Cancer | TCE | 1.18 | 0.33 | 4.19 | 0.80 | 0.96 | 0.27 | 3.37 | 0.95 |
| Colon Cancer | TCE | 1.00 | 0.26 | 3.84 | 1.00 | 0.52 | 0.12 | 2.34 | 0.39 |
| Breast Cancer | TCE | 1.14 | 0.43 | 3.04 | 0.80 | 0.80 | 0.23 | 2.78 | 0.72 |
| Prostate Cancer | TCE | 2.55 | 0.26 | 25.15 | 0.42 | 2.39 | 0.27 | 21.14 | 0.43 |
| **Smoking-related Diseases (not known to be related to solvent exposure):** | | | | | | | | | |
| COPD | TCE | 0.66 | 0.29 | 1.52 | 0.33 | 0.61 | 0.29 | 1.27 | 0.19 |
| Cardiovascular Disease | TCE | 0.72 | 0.50 | 1.05 | 0.09 | 0.81 | 0.58 | 1.13 | 0.22 |
|  |  |  |  |  |  |  |  |  |  |
| Underlying Cause | Contaminant | Medium Exposure | 95% LCL | 95% UCL | P-value | High Exposure | 95% LCL | 95% UCL | P-value |
| All Cancers | PCE | 0.94 | 0.68 | 1.32 | 0.74 | 0.90 | 0.65 | 1.25 | 0.54 |
| **Diseases of Primary Interest**: | | | | | | | | | |
| Hematopoietic Cancers | PCE | 0.69 | 0.22 | 2.23 | 0.54 | 0.89 | 0.33 | 2.37 | 0.81 |
| Leukemia | PCE | 1.00 | 0.14 | 7.39 | 1.00 | 1.82 | 0.36 | 9.32 | 0.47 |
| **Diseases of Secondary Interest**: | | | | | | | | | |
| Lung Cancer | PCE | 1.06 | 0.60 | 1.86 | 0.84 | 0.82 | 0.47 | 1.43 | 0.50 |
| Pancreatic Cancer | PCE | 0.28 | 0.05 | 1.39 | 0.12 | 0.52 | 0.14 | 1.90 | 0.32 |
| Colorectal Cancer | PCE | 1.19 | 0.34 | 4.22 | 0.79 | 1.03 | 0.30 | 3.62 | 0.96 |
| Colon Cancer | PCE | 1.01 | 0.26 | 3.86 | 0.99 | 0.56 | 0.12 | 2.50 | 0.44 |
| Breast Cancer | PCE | 1.24 | 0.46 | 3.30 | 0.67 | 0.89 | 0.25 | 3.09 | 0.85 |
| Prostate Cancer | PCE | 3.46 | 0.38 | 31.65 | 0.27 | 2.08 | 0.23 | 18.91 | 0.52 |
| **Smoking-related Diseases (not known to be related to solvent exposure):** | | | | | | | | | |
| COPD | PCE | 0.56 | 0.24 | 1.31 | 0.18 | 0.61 | 0.30 | 1.27 | 0.19 |
| Cardiovascular Disease | PCE | 0.70 | 0.49 | 1.01 | 0.06 | 0.79 | 0.57 | 1.10 | 0.16 |

Excluded because causes of death < 10: kidney cancer, bladder cancer, liver cancer, esophageal cancer, Hodgkin lymphoma, non-Hodgkin lymphoma, multiple myeloma, brain cancer, rectal cancer, soft tissue sarcoma, oral cancers, laryngeal cancer, cervical cancer, liver and kidney diseases, Parkinson’s disease, ALS, multiple sclerosis, and stomach cancer.

Models were adjusted by sex, race, occupation (blue collar vs white collar) and education level.

TVOC: total contaminants (PCE, TCE, t-1,2-dichloroethylene, VC, and benzene)

VC: vinyl chloride TCE: trichloroethylene

PCE: tetrachloroethylene

**Table S2b: Adjusted Hazard ratios for categorized Cumulative Exposures (<median (ref.), ≥median) 10 year lag. Camp Lejeune cohort (N=4,647)**

| **Underlying Cause of Death** | **Exposure** | **Hazard Ratio** | **95% LCL** | **95% UCL** | **P-value** |
| --- | --- | --- | --- | --- | --- |
| All Cancers | TVOC | **0.88** | 0.67 | 1.15 | 0.34 |
| **Diseases of Primary Interest**: | | | | | |
| Kidney Cancer | TVOC | **4.44** | 0.52 | 38.19 | 0.17 |
| Esophageal Cancer | TVOC | **2.43** | 0.24 | 24.87 | 0.45 |
| Hematopoietic Cancers | TVOC | **0.88** | 0.37 | 2.10 | 0.78 |
| Multiple Myeloma | TVOC | **0.60** | 0.11 | 3.23 | 0.56 |
| Leukemia | TVOC | **1.37** | 0.35 | 5.35 | 0.65 |
| Non-Hodgkin Lymphoma | TVOC | **0.32** | 0.05 | 2.11 | 0.24 |
| **Diseases of Secondary Interest**: | | | | | |
| Brain Cancer | TVOC | **1.05** | 0.22 | 5.01 | 0.95 |
| Lung Cancer | TVOC | **0.75** | 0.48 | 1.18 | 0.21 |
| Laryngeal Cancer | TVOC | **0.25** | 0.02 | 2.45 | 0.23 |
| Pancreatic Cancer | TVOC | **0.52** | 0.16 | 1.66 | 0.27 |
| Breast Cancer | TVOC | **1.21** | 0.50 | 2.95 | 0.68 |
| Prostate Cancer | TVOC | **1.05** | 0.26 | 4.34 | 0.95 |
| Colorectal Cancer | TVOC | **0.79** | 0.28 | 2.22 | 0.66 |
| Rectal Cancer | TVOC | **1.78** | 0.17 | 18.58 | 0.63 |
| Colon Cancer | TVOC | **0.63** | 0.19 | 2.08 | 0.44 |
| Oral Cancers | TVOC | **2.29** | 0.22 | 23.53 | 0.48 |
| Kidney Disease | TVOC | **0.74** | 0.16 | 3.47 | 0.71 |
| Liver Disease | TVOC | **0.54** | 0.13 | 2.31 | 0.40 |
| Parkinson’s Disease | TVOC | **2.52** | 0.21 | 30.83 | 0.47 |
| **Smoking-related Diseases (not known to be related to solvent exposure):** | | | | | |
| Stomach Cancer | TVOC | **0.31** | 0.03 | 3.46 | 0.34 |
| COPD | TVOC | **0.56** | 0.30 | 1.06 | 0.08 |
| Cardiovascular Disease | TVOC | **0.89** | 0.66 | 1.18 | 0.41 |
|  |  |  |  |  |  |
| All Cancers | benzene | **0.91** | 0.69 | 1.20 | 0.50 |
| **Diseases of Primary Interest**: | | | | | |
| Kidney Cancer | benzene | **1.82** | 0.34 | 9.78 | 0.49 |
| Esophageal Cancer | benzene | **2.27** | 0.22 | 23.32 | 0.49 |
| Hematopoietic Cancers | benzene | **0.87** | 0.37 | 2.08 | 0.76 |
| Multiple Myeloma | benzene | **0.55** | 0.10 | 3.00 | 0.49 |
| Leukemia | benzene | **1.38** | 0.35 | 5.43 | 0.64 |
| Non-Hodgkin Lymphoma | benzene | **0.33** | 0.05 | 2.14 | 0.24 |
| **Diseases of Secondary Interest**: | | | | | |
| Brain Cancer | benzene | **1.11** | 0.23 | 5.34 | 0.90 |
| Lung Cancer | benzene | **0.79** | 0.50 | 1.25 | 0.32 |
| Laryngeal Cancer | benzene | **0.26** | 0.03 | 2.54 | 0.25 |
| Pancreatic Cancer | benzene | **0.37** | 0.11 | 1.26 | 0.11 |
| **Underlying Cause of Death** | **Exposure** | **Hazard Ratio** | **95% LCL** | **95% UCL** | **P-value** |
| Breast Cancer | benzene | **1.28** | 0.53 | 3.12 | 0.58 |
| Prostate Cancer | benzene | **1.85** | 0.37 | 9.27 | 0.45 |
| Colorectal Cancer | benzene | **0.78** | 0.28 | 2.20 | 0.64 |
| Rectal Cancer | benzene | **1.80** | 0.17 | 18.63 | 0.62 |
| Colon Cancer | benzene | **0.61** | 0.18 | 2.02 | 0.41 |
| Oral Cancers | benzene | **2.33** | 0.23 | 23.86 | 0.48 |
| Kidney Disease | benzene | **0.43** | 0.09 | 2.08 | 0.30 |
| Liver Disease | benzene | **0.52** | 0.12 | 2.23 | 0.38 |
| Parkinson’s Disease | benzene | **2.52** | 0.20 | 31.59 | 0.47 |
| **Smoking-related Diseases (not known to be related to solvent exposure):** | | | | | |
| Stomach Cancer | benzene | **1.31** | 0.16 | 10.91 | 0.80 |
| COPD | benzene | **0.57** | 0.30 | 1.08 | 0.08 |
| Cardiovascular Disease | benzene | **0.82** | 0.62 | 1.10 | 0.19 |
|  |  |  |  |  |  |
| All Cancers | VC | **0.97** | 0.74 | 1.27 | 0.80 |
| **Diseases of Primary Interest**: | | | | | |
| Esophageal Cancer | VC | **2.41** | 0.24 | 24.65 | 0.46 |
| Hematopoietic Cancers | VC | **0.93** | 0.39 | 2.20 | 0.87 |
| Multiple Myeloma | VC | **0.61** | 0.11 | 3.24 | 0.56 |
| Leukemia | VC | **1.44** | 0.37 | 5.63 | 0.60 |
| Non-Hodgkin Lymphoma | VC | **0.36** | 0.05 | 2.32 | 0.28 |
| **Diseases of Secondary Interest**: | | | | | |
| Brain Cancer | VC | **0.56** | 0.10 | 3.07 | 0.50 |
| Lung Cancer | VC | **0.88** | 0.56 | 1.39 | 0.58 |
| Laryngeal Cancer | VC | **0.25** | 0.03 | 2.51 | 0.24 |
| Pancreatic Cancer | VC | **0.54** | 0.17 | 1.74 | 0.30 |
| Breast Cancer | VC | **1.29** | 0.53 | 3.13 | 0.58 |
| Prostate Cancer | VC | **1.13** | 0.28 | 4.62 | 0.86 |
| Colorectal Cancer | VC | **0.83** | 0.30 | 2.32 | 0.72 |
| Colon Cancer | VC | **0.44** | 0.13 | 1.52 | 0.19 |
| Oral Cancers | VC | **2.44** | 0.24 | 24.82 | 0.45 |
| Kidney Disease | VC | **0.46** | 0.10 | 2.20 | 0.33 |
| Liver Disease | VC | **0.55** | 0.13 | 2.37 | 0.42 |
| Parkinson’s Disease | VC | **2.81** | 0.23 | 34.11 | 0.42 |
| **Smoking-related Diseases (not known to be related to solvent exposure):** | | | | | |
| Stomach Cancer | VC | **1.25** | 0.15 | 10.42 | 0.84 |
| COPD | VC | **0.54** | 0.29 | 1.03 | 0.06 |
| Cardiovascular Disease | VC | **0.90** | 0.68 | 1.20 | 0.48 |
|  |  |  |  |  |  |
| All Cancers | TCE | **0.89** | 0.68 | 1.17 | 0.42 |
| **Diseases of Primary Interest**: | | | | | |
| Esophageal Cancer | TCE | **2.41** | 0.23 | 24.63 | 0.46 |
| **Underlying Cause of Death** | **Exposure** | **Hazard Ratio** | **95% LCL** | **95% UCL** | **P-value** |
| Hematopoietic Cancers | TCE | **0.87** | 0.37 | 2.07 | 0.76 |
| Multiple Myeloma | TCE | **0.60** | 0.11 | 3.18 | 0.55 |
| Leukemia | TCE | **1.36** | 0.35 | 5.30 | 0.66 |
| Non-Hodgkin Lymphoma | TCE | **0.32** | 0.05 | 2.10 | 0.24 |
| **Diseases of Secondary Interest**: | | | | | |
| Brain Cancer | TCE | **1.02** | 0.21 | 4.86 | 0.98 |
| Lung Cancer | TCE | **0.73** | 0.47 | 1.16 | 0.18 |
| Laryngeal Cancer | TCE | **0.24** | 0.02 | 2.37 | 0.22 |
| Pancreatic Cancer | TCE | **0.50** | 0.16 | 1.61 | 0.25 |
| Breast Cancer | TCE | **1.17** | 0.48 | 2.84 | 0.74 |
| Prostate Cancer | TCE | **1.87** | 0.38 | 9.32 | 0.44 |
| Colorectal Cancer | TCE | **0.78** | 0.28 | 2.18 | 0.64 |
| Rectal Cancer | TCE | **1.75** | 0.17 | 18.17 | 0.64 |
| Colon Cancer | TCE | **0.62** | 0.19 | 2.04 | 0.43 |
| Oral Cancers | TCE | **2.21** | 0.22 | 22.72 | 0.50 |
| Kidney Disease | TCE | **0.74** | 0.16 | 3.44 | 0.70 |
| Liver Disease | TCE | **0.53** | 0.12 | 2.27 | 0.39 |
| Parkinson’s Disease | TCE | **2.51** | 0.21 | 30.76 | 0.47 |
| **Smoking-related Diseases (not known to be related to solvent exposure):** | | | | | |
| Stomach Cancer | TCE | **0.30** | 0.03 | 3.35 | 0.33 |
| COPD | TCE | **0.55** | 0.29 | 1.05 | 0.07 |
| Cardiovascular Disease | TCE | **0.89** | 0.67 | 1.19 | 0.44 |
|  |  |  |  |  |  |
| All Cancers | PCE | **1.04** | 0.79 | 1.37 | 0.80 |
| **Diseases of Primary Interest**: | | | | | |
| Esophageal Cancer | PCE | **2.12** | 0.21 | 21.85 | 0.53 |
| Hematopoietic Cancers | PCE | **0.83** | 0.35 | 1.97 | 0.68 |
| Multiple Myeloma | PCE | **0.55** | 0.10 | 2.92 | 0.48 |
| Leukemia | PCE | **1.30** | 0.33 | 5.05 | 0.71 |
| Non-Hodgkin Lymphoma | PCE | **0.33** | 0.05 | 2.11 | 0.24 |
| **Diseases of Secondary Interest**: | | | | | |
| Brain Cancer | PCE | **0.93** | 0.19 | 4.47 | 0.93 |
| Lung Cancer | PCE | **1.01** | 0.64 | 1.62 | 0.95 |
| Laryngeal Cancer | PCE | **0.20** | 0.02 | 2.08 | 0.18 |
| Pancreatic Cancer | PCE | **0.46** | 0.14 | 1.49 | 0.20 |
| Breast Cancer | PCE | **1.26** | 0.52 | 3.07 | 0.61 |
| Prostate Cancer | PCE | **1.87** | 0.38 | 9.19 | 0.44 |
| Colorectal Cancer | PCE | **0.96** | 0.34 | 2.69 | 0.93 |
| Colon Cancer | PCE | **0.57** | 0.17 | 1.88 | 0.36 |
| Oral Cancers | PCE | **2.03** | 0.20 | 20.76 | 0.55 |
| Kidney Disease | PCE | **0.43** | 0.09 | 2.04 | 0.29 |
| Liver Disease | PCE | **0.49** | 0.11 | 2.10 | 0.34 |
| Parkinson’s Disease | PCE | **2.68** | 0.22 | 33.28 | 0.44 |
| **Underlying Cause of Death** | **Exposure** | **Hazard Ratio** | **95% LCL** | **95% UCL** | **P-value** |
| **Smoking-related Diseases (not known to be related to solvent exposure):** | | | | | |
| Stomach Cancer | PCE | **1.01** | 0.12 | 8.50 | 0.99 |
| COPD | PCE | **0.60** | 0.32 | 1.13 | 0.11 |
| Cardiovascular Disease | PCE | **0.86** | 0.65 | 1.15 | 0.32 |

Diseases excluded because of small numbers included: bladder cancer, liver cancer, Hodgkin’s lymphoma, soft tissue sarcoma, ALS, and multiple sclerosis. In addition, rectal cancer and kidney cancer were excluded from some tables due to small numbers.

Models were adjusted by sex, race, occupation (blue collar vs white collar) and education level.

TVOC: total contaminants (PCE, TCE, t-1,2-dichloroethylene, VC, and benzene)

VC: vinyl chloride TCE: trichloroethylene

PCE: tetrachloroethylene
