# Supplementary material for: Mortality study of civilian employees exposed to contaminated drinking water at USMC Base Camp Lejeune: a retrospective cohort study
Source: Environ Health. 2014 Aug 13;13:68. doi: 10.1186/1476-069X-13-68 (PMC4237831; doi:10.1186/1476-069X-13-68)

Figure S1a.1 Spline of leukemias and cumulative exposure to PCE


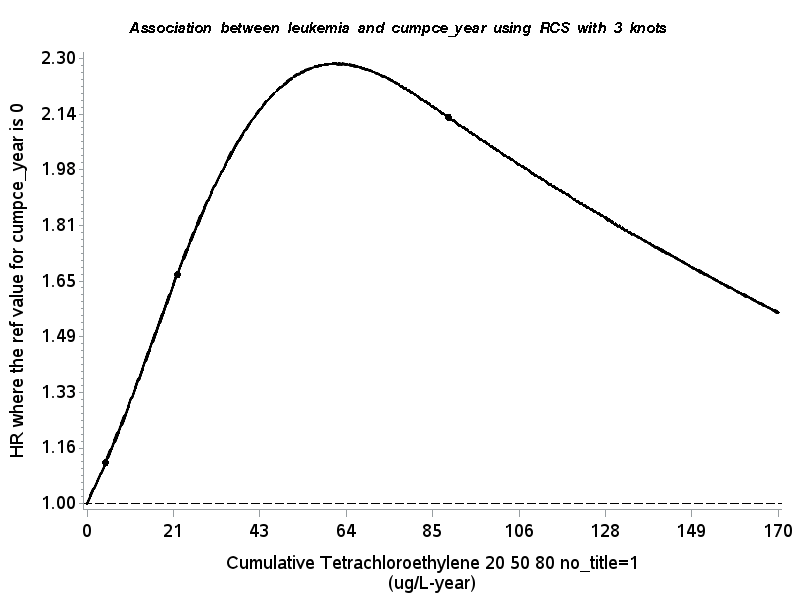


Knot #1: 4.6 µg/L-year (20^th^ percentile)

Knot #2: 22.3 µg/L-year (50^th^ percentile)

Knot #3: 88.8 µg/L-year (80^th^ percentile)

Figure S1a.2 Spline of leukemias and cumulative exposure to PCE with 95% confidence interval bands.


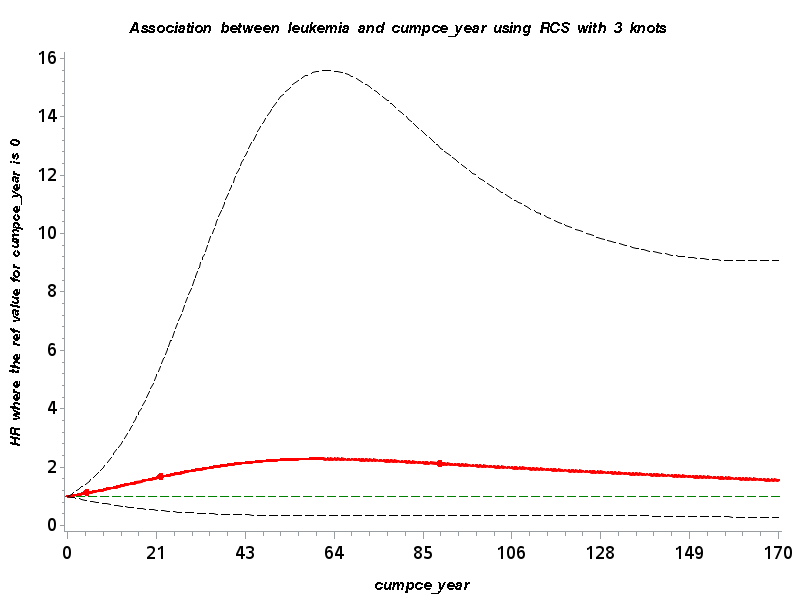


Figure S1b.1 Spline of leukemias and cumulative exposure to vinyl chloride


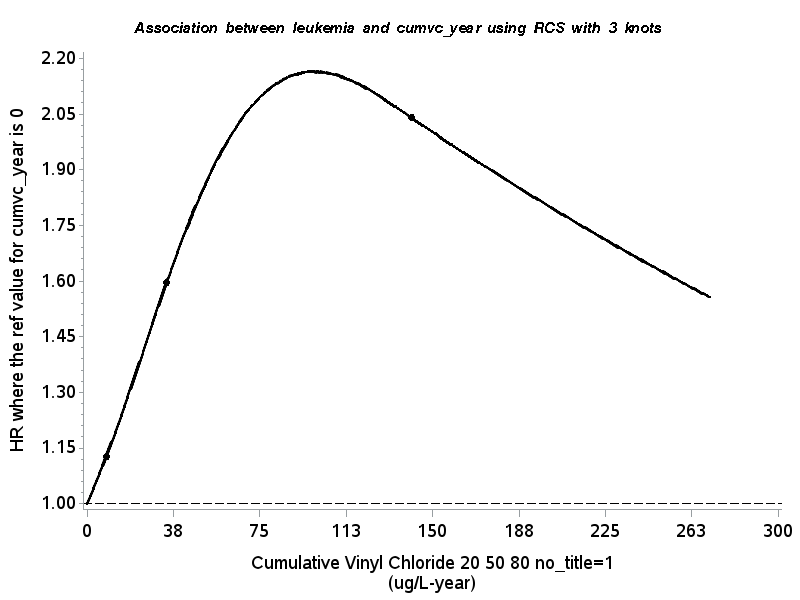


Knot #1: 8.41 µg/L-year (20^th^ percentile)

Knot #2: 34.54 µg/L-year (50^th^ percentile)

Knot #3: 140.87 µg/L-year (80^th^ percentile)

Figure S1b.2 Spline of leukemias and cumulative exposure to vinyl chloride with 95% confidence interval bands.


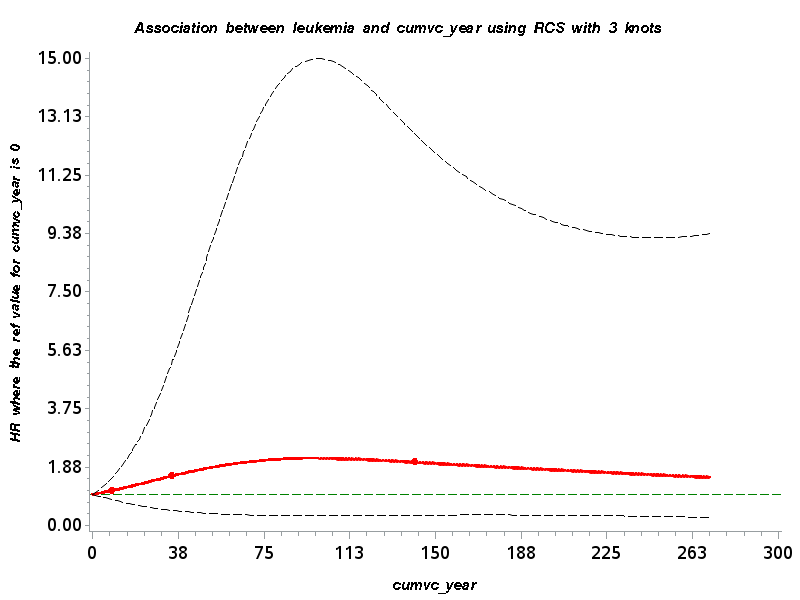

Supplement: Additional file 4 — 1 Spline of leukemias and cumulative exposure to PCE and vinyl chloride. [file 1476-069X-13-68-S4.docx]
